# Guided Water Percolation in 3D-Printed Gas Diffusion Layers for Polymer Electrolyte Fuel Cells

**DOI:** 10.1021/acsami.5c00770

**Published:** 2025-04-10

**Authors:** Tim Dörenkamp, Ambra Zaccarelli, Felix N. Büchi, Thomas J. Schmidt, Jens Eller

**Affiliations:** † PSI Center for Energy and Environmental Sciences, Villigen PSI CH-5232, Switzerland; ‡ Department of Materials, ETH Zürich, Zürich CH-8093, Switzerland; § Institute of Molecular Physical Science, ETH Zürich, Zürich CH-8093, Switzerland

**Keywords:** PEFC, water transport, GDL, 3D-printing, X-ray radiography, X-ray tomography

## Abstract

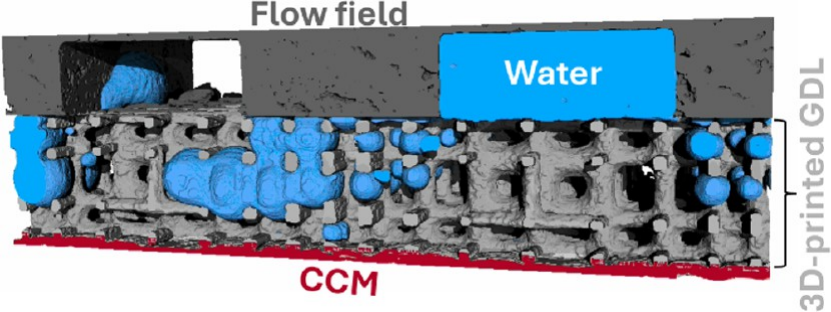

The accumulation of liquid water in the gas diffusion layer (GDL) and associated clogging of the reactant pathways are limiting factors for the performance of polymer electrolyte fuel cells (PEFC). The design and manufacturing of GDLs with a deterministic pore space have the potential to accelerate the development of next-generation PEFC with an optimized balance between reactant supply and product removal. In this study, we explore the potential of GDLs with tailored pore structures obtained from the carbonization of a 3D-printed precursor. Three different GDL designs are investigated by using operando X-ray radiography and subsequent X-ray tomography to track the water pathways. The results confirm the effectiveness of the designed features in terms of controlled liquid water percolation and reveal a trend toward vapor phase transport rather than liquid transport of water away from the catalyst layer interface along with a strong convective flow within the highly porous ordered structures.

## Introduction

The urgent need to reduce CO_2_ emissions has recently accelerated efforts to develop clean energy systems. Among others, electrochemical energy conversion technologies, such as electrolyzers and fuel cells, are promising solutions to decarbonize the energy sector by converting green electricity into hydrogen, which can be stored, transported, and later reconverted into electric power. Over the past decades, polymer electrolyte fuel cells (PEFC) have emerged as an auspicious candidate to replace fossil fuel powered engines in vehicles, in particular in the heavy-duty sector.^1^

PEFC convert hydrogen and oxygen into electricity, with water and heat as the only byproducts. The hydrogen is supplied via flow fields on the anode side of the cell, where it diffuses through a gas diffusion layer (GDL) toward the catalyst layer (CL) and is split into protons and electrons. Oxygen is supplied to the cathode flow field and, similarly to the anode side, evenly distributed to the CL by a GDL. The anode and cathode compartments are separated by a polymer electrolyte membrane, such as Nafion. Besides the transport of reactants to the CL, the GDL has several important functions. It must ensure electrical contact between the bipolar plate and the electrode, transport waste heat from the electrochemical reaction to the cooling channels, and at the cathode remove the product water toward the flow field where it is dragged away with the feed gas and removed from the stack. Proper water management is important for the PEFC performance. On the one hand, the membrane needs to remain hydrated to keep a high protonic conductivity, and on the other hand, the product water needs to be efficiently removed through the GDL to maintain low water saturation in the porous layers to avoid flooding and the associated reactant starvation. State of the art GDLs are thin clothes, felts, or papers with a thickness of ∼140–300 μm made of carbon fibers with a diameter of ∼7–10 μm manufactured from a carbonized PAN precursor. They are typically treated with a hydrophobic polytetrafluoroethylene (PTFE) coating to improve hydrophobicity.^2^ In addition, GDLs feature a microporous layer (MPL) to reduce the contact resistance and mechanical stress on the CL and promote back-diffusion of product water from the cathode to the anode, which helps to keep the membrane hydrated.^3−5^ Due to its constricted number of breakthrough points, the MPL also helps to reduce the saturation of liquid water within the GDL,^6,7^ while its lower thermal conductivity leads to an increased temperature and consequently lower liquid saturation next to the CL.^8^

Data from noninvasive imaging techniques, such as X-ray radiography and X-ray tomography, offer a comprehensive understanding of water transport phenomena in PEFC. At high temporal resolutions, key phenomena such as initial water breakthrough points, Haines jumps, and transport path breakdown could be observed.^9^ Kato et al. provided mechanistic insights into the water transport in operating PEFC across various conditions by employing X-ray radiography. Depending on the operating temperature and relative humidity of the supply gases, they found four distinct modes, namely: concurrent liquid and vapor transport, dominating liquid transport, vapor transport, and vapor transport to the ribs with only condensation near the ribs.^10^ For PEFC operation, liquid water removal from the CL is particularly critical as it can block the pores of the GDL and consequently hinders the diffusion of oxygen toward the CL resulting in mass transport losses.^11^ It has become common knowledge in the community that liquid water percolation in PEFC is dominated by capillary forces where water follows the path of the lowest resistance according to the Young–Laplace law, which describes the relationship between the breakthrough pressure of water pushing through a throat and the contact angle as well as the throat radius.^12,13^ It has been shown that the stochastic pore structure of the GDL significantly influences the percolation path, in a majority of cases with an undesired extent of in-plane percolation.^14,15^ The effect of uncontrolled water percolation is amplified by the heterogeneous contact angle distribution due to the uneven PTFE distribution.^16,17^

Several attempts have been made to alter the structural or wettability properties of GDLs to improve their water discharge behavior. Forner-Cuenca et al. proposed a method to introduce hydrophilic lines into the hydrophobic GDL, achieving improved water removal while simultaneously creating low saturation regions for gas transport.^18^ Wen et al. prepared a Janus GDL via layer-by-layer filtration and laser drilling, which demonstrated superior antiflooding capabilities while increasing the peak power density.^19^ Additionally, Csoklich et al. found improved performance and water management in GDLs adding slits via laser perforation.^20,21^ They also replaced the conventional cathode GDL by an entirely deterministic woven material and could obtain significantly reduced mass transport losses and enhanced fuel cell performance, especially at lower temperatures as obtained during startup.^22^ Finally, Niblett et al. successfully utilized a GDL with an ordered pore structure derived from the carbonization of a 3D-printed polymer precursor, paving the way for next generation GDL with tailored morphologies.^23^

In this work, we employ digital light processing (DLP) 3D printing and subsequent carbonization to manufacture GDLs with different deterministic pore structures for guided liquid water transport during PEFC operation. The structures are probed by using *operando* X-ray radiography as well as X-ray tomography to track the water percolation pathways after a current jump.

## Methodology

### Sample Preparation and Characterization

The GDLs were designed using Autodesk Inventor and converted into STL. The STL files were prepared for 3D printing using the CHITUBOX slicer. 3D printing was done in a Phrozen Mini 8K desktop printer with a pixel resolution of 22 × 22 μm and a layer thickness of 10 μm. The exposure time for each layer was set to 1.3 s resulting in a printing time of ∼7 h for sample dimensions of 7.4 mm width × 1.1 mm depth × 27.5 mm height, where the latter is the printing direction. Note that multiple samples were printed simultaneously, which dramatically reduces the effective printing time per specimen. The resin used was the photocurable Phrozen Aqua-Gray 8K. The composition of the resin can be found in Table S1.

**Figure 1:**
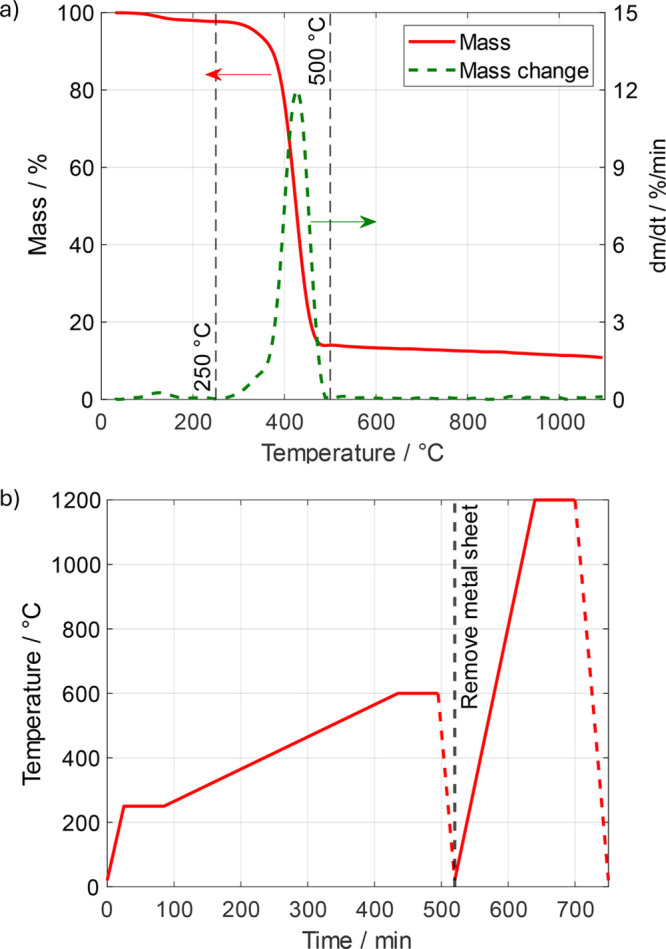
a) Thermogravimetry analysis of printed Phrozen Aqua 8K resin. b) Two step temperature ramp used for carbonization in a tube furnace.

After printing the structures were carbonized in a tube furnace (Carbolite Gero GmbH & Co. KG) with a nitrogen flow of 250 mL/min to obtain an inert atmosphere. A thermogravimetric analysis (TGA) was done to identify the critical temperature range in which pyrolysis takes place (Figure 1a). The peak observed around 130 °C is likely a result of the evaporation of water or solvents.^23^ Based on TGA, a two-step temperature ramp for carbonization was implemented, as shown in Figure 1b. In the first step, the printed structure was covered by a porous metal sheet to mitigate deformation associated with the degradation of the sample. In a second step, the metal sheet was removed, and the final temperature increased to 1200 °C to improve the electrical conductivity of the carbon. For the second step, no further deformations were expected according to the results of the TGA.

Hydrophobic treatment of the carbonized samples was achieved by drop casting a solution of 0.1 wt % amorphous fluoropolymer (Teflon AF 2400) in Fluorinert FC-70 and drying it in a vacuum oven at a temperature of 80 °C for 1 h. The solution was obtained by mixing the Teflon particles with the solvent and stirring it at a temperature of 70 °C until a clear solution was obtained.^24^

Scanning electron microscopy (SEM) was done with a Carl Zeiss Ultra55 instrument to analyze the structural surface properties of the carbonized specimen. Furthermore, energy-dispersive X-ray spectroscopy (EDS) was performed with an EDAX APOLLO XV Silicon Drift Detector to study the surface composition to confirm the success of the hydrophobic treatment. The acceleration voltage for SEM and EDS was set to 3 and 14 keV, respectively. The electrical conductivity was obtained by using a 4-point probe.

### Fuel Cell Testing

**Figure 2:**
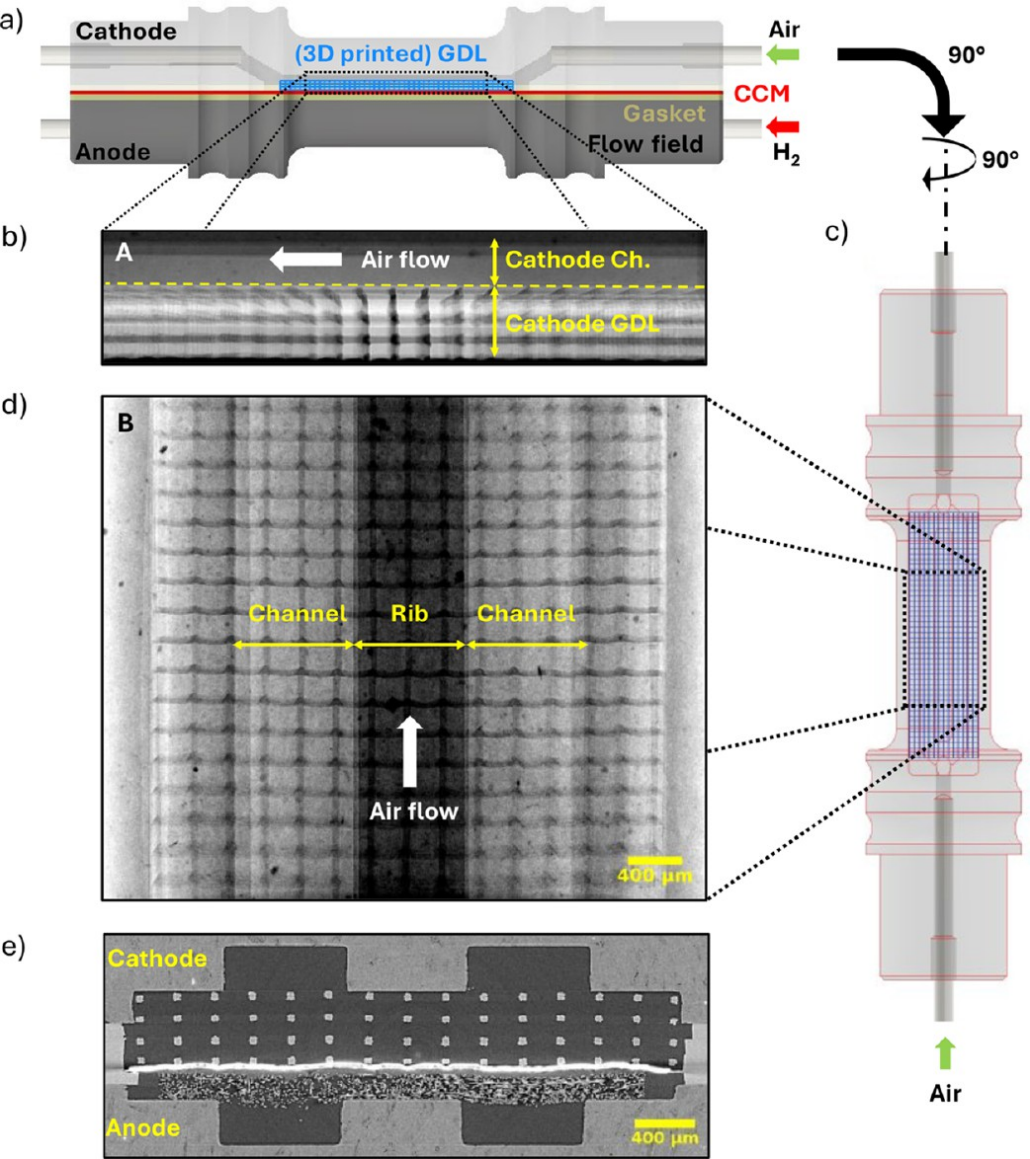
a) Schematic of the cell assembly used for operando CT diagnostics. b) Radiographic projection of the dry cathode GDL and channel in the direction perpendicular to CL (perspective “A”). c) Cell scheme tilted and rotated by 90° to the right with respect to (a). d) Radiographic projection of the dry cell picturing the channel-rib (perspective “B”). e) Tomographic image slice exemplarily showing the cross section for one of the cell assemblies.

The 3D-printed and carbonized structures were assembled as cathode GDLs together with a catalyst coated membrane (CCM) (Gore Primea A510.1/M815.15/C510.4 with a 15 μm thick reinforced Gore-Select membrane and anode/cathode Pt loadings of 0.1/0.4 mg/cm^2^) and a Freudenberg H2315 C2 anode GDL to form the membrane electrode assembly (MEA). For the Toray reference, two TPH-090 GDLs with a 10% PTFE coating were stacked at the cathode. For the Freudenberg reference, the cathode GDL was the same as that of the anode. The CCM was laser ablated to form a defined active area of 0.16 cm^2^. Laser ablation was performed at LPKF Laser Electronics SE (30827 Garbsen, Germany). The MEA was then sandwiched between two double channel graphite flow fields (BMA5, Eisenhuth, Germany) specifically designed for X-ray investigations.^25^ Free-standing MPLs with a thickness of 34 μm have been prepared according to the procedure described by Simon et al.^26^ On both sides, fluoroethylene propylene (FEP) gaskets were used to seal the cell against the environment. On the cathode side, the thickness of the gaskets was adjusted according to the thickness of the incompressible 3D-printed GDL. On the anode side, a 75 μm gasket was used yielding a compression of the Freudenberg GDL of 25%. A scheme of the cell assembly is shown in Figure 2a,c, and a cross sectional view of the cell assembly obtained from X-ray tomography is shown in Figure 2e.

For the current jump experiment, the cells were dried with 172 mL/min N_2_/N_2_ at RH 70%/70% for at least 5 min until the high frequency resistance formed a plateau. After drying, the cell was flushed with oversaturated active gases H_2_/air until open circuit voltage (OCV) was reached. For the radiography, the cell was operated in galvanostatic mode with a hold time of 10 min to reach steady state.^7^ After each radiography, the gas flow as well as the cell heating were turned off and the current density was set to zero to “freeze” the water content for subsequent tomographic imaging.^27^ The conditions tested during radiographic imaging are listed in Table 1. For each radiography orientation, the drying and current jump sequence as well as the final CT scan were repeated. Polarization curves were recorded in constant voltage mode starting from OCV down to 400 mV in 50 mV steps. Each voltage step was held for 30 s and the values were averaged to obtain the corresponding data points for the polarization curve. The high frequency resistance (HFR) was measured at a frequency of 1 kHz using a Tsuruga milliohmmeter. For the polarization curves, the flow rate was set to 173 mL/min on both the anode and cathode, resulting in a channel flow velocity of 6 m/s.

**Table 1:** Operating Conditions for Operando and In Situ CT Experiments

Current dens. [A/cm^2^]	Temperature [°C]	Humidity [%]	Flow rate [NmL/min]	Pressure [bar_abs_]
0.5	30	An/Cat: 110/110	An/Cat: 173/57	1
1.5	50	An/Cat: 110/110	An/Cat: 173/57	1

### Image Acquisition

The cells were mounted in an in-house designed sample holder on the rotation stage of a CT scanner (Phoenix nanotom m, General Electric, Germany). The acceleration voltage of the X-ray tube was set to 80 kV and the current was set to 230 μA. Radiographic images were recorded during the current jump experiments out of two different perspectives as shown in Figure 2b,d to learn about the dynamic water growth with respect to the distance from the Cl and the channel-rib region, respectively. Later, the two perspectives shown in Figure 2b,d will be termed perspective “A” and perspective “B”, respectively. A series of 1200 projections with an exposure time of 500 ms was collected for each condition and perspective. Note that the perspective shown in Figure 2e cannot be analyzed using radiographic imaging due to the cell’s mounting configuration within the CT. The magnification was 33.3, leading to a pixel size of about 3 μm in the center of the beam. Tomographic imaging was done using the fast scan option to achieve scan times of 10 min with 1200 projections at an exposure time of 500 ms. For high quality dry scans to obtain solid masks of the cell, 2000 projections were recorded each being an average of 3 exposures of 500 ms per projection with one projection skipped per image resulting in a scan time of 67 min.

### Image Processing

To obtain the qualitative water signal of the radiographic time series of the current jump experiment, the first acquired image served as a reference and was subtracted from all the other images. Therefore, the remaining change in grayscale over time is associated with growth or relocation of liquid water. The tomographic data were processed to receive the liquid volume fraction (LVF) at the end of each current jump. The dry scans were aligned to the Cartesian coordinates by using ImageJ. The wet scans were then registered to the reference dry scans by using SimpleElastix.^28^ Water masks have been extracted by subtracting the reference dry scan from the aligned wet scans and using a coarse 3D median filter (*R* = 3) followed by a 2D median (*R* = 5) filter for denoising and subsequent Otsu thresholding.

### Simulation

**Figure 3:**
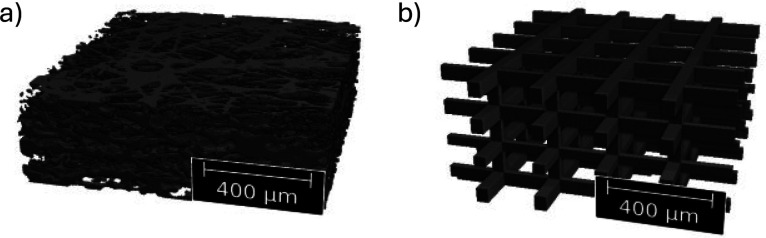
3D rendering of the structures used for the permeability and diffusivity simulation for the a) Toray and b) 3DTP.

Numerical simulations of the permeability and the relative diffusivity of the printed GDL samples as well as the estimated gas flow velocity in the flow channels and GDLs during operation were performed in a GeoDict 2022 instrument (Math2Market, Germany). The permeability and diffusivity were calculated for the 3DTP by using the SatuDict plugin with periodic boundary conditions in the through-plane direction. The gas flow simulations for all 3D-printed designs as well as the Toray reference were performed by using the Stokes solver in the FlowDict module with the inlet flow rate of 57 mL/min and periodic boundary conditions. Through-plane velocity profiles were obtained for the rib and channel domain by averaging both along and perpendicular to the channel direction. The 3D-printed GDL structure as well as the cell assembly were modeled in Autodesk Inventor and converted into STL. Import Geo-CAD was used to import the assemblies in GeoDict. For the Toray reference case, the segmented tomographic data were used and imported with Import Geo-Vol. 3D renderings of the corresponding structures are shown in Figure 3.

## Results and Discussion

### Carbonization and Wettability

**Figure 4:**
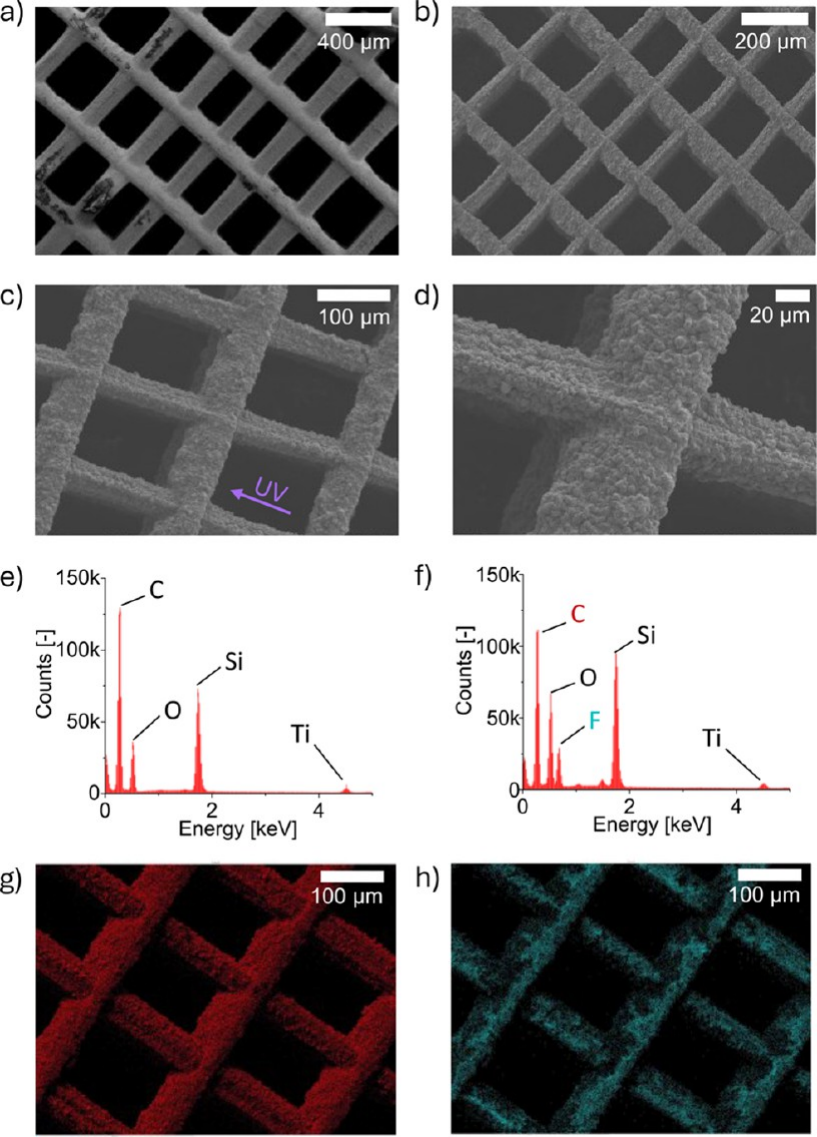
a) SEM image of the 3D-printed structure before carbonization. b) SEM image of 3D- printed structure after carbonization. c,d) SEM image of the carbonized structure at different resolutions. e) EDS spectra of the structure with hydrophobic coating. f) EDS spectra of the structure without hydrophobic coating. g) EDS map of the carbon signal of the coated sample. h) EDS map of the fluorine signal of the coated sample.

The assessment of the carbonized samples by SEM reveals that the structural properties have successfully been maintained despite severe mass loss and associated volume shrinkage (see Figure 4a,b). The heterogeneous thickness of the pillars is caused by the overcuring effect in the UV-light exposure direction as depicted in Figure 4c. The smallest achieved pillar sizes are obtained in a perpendicular orientation with respect to the LCD screen and found to be ∼ 40 μm. The pyrolytic carbon looks like an agglomeration of carbon particles at the size of several 100 nm to a few micrometers, which leads to a very high surface roughness (see Figure 4d). This could be of further interest for non-PEFC applications where carbon acts as a catalyst and could profit from an increased electrochemical surface area (ECSA).^29,30^

EDS spectra of the carbonized structure before and after hydrophobic treatment show the appearance of a clear fluorine signal for the hydrophobized sample (see Figure 4e,f, respectively). Besides the carbon and fluorine signals, the EDS reveals the presence of silicon, oxygen, and titanium. This is justified by the resin formula which contains both silicon and titanium (see Table S1). Both are likely to be present as solid oxides and are therefore not volatile during the pyrolysis. The distribution of the different phases within the material revealed by cross-sectional SEM/EDS measurements is shown in Figure S1. No negative effects on the cell performance are expected for the short-term operation, as conducted in this study. However, it has been observed that decomposition products of silicone seals which are in contact with the membrane can contribute to catalyst poisoning as well as altering of the wettability properties.^31^ This would need to be considered for performance assessments on longer time scales. The small peak at around 1.5 keV in Figure 4f is most likely a k-edge signal of aluminum, which is the material of the sample holder used for SEM and EDS measurements and therefore not part of the sample composition.

The EDS maps of the carbon signal and the fluorine signal are shown in Figure 4e,f, respectively. It confirms that the coating method results in a homogeneous hydrophobic layer. The contact angle of the coating was found to be ∼113°.^32^ Conventionally, hydrophobicity is achieved by dip-coating the carbon fiber material into a suspension with polytetrafluoroethylene (PTFE) followed by a heat treatment to adhere the PTFE particles to the carbon. This usually leads to heterogeneous wettability properties of the structures due to an uneven distribution of the PTFE.^2,12^ In the scope of this study, it is important to mitigate the effect of heterogeneous surface wettability to ensure that water percolation is determined only by the morphological properties, which is why a different coating approach was used.

### GDL Designs

**Figure 5:**
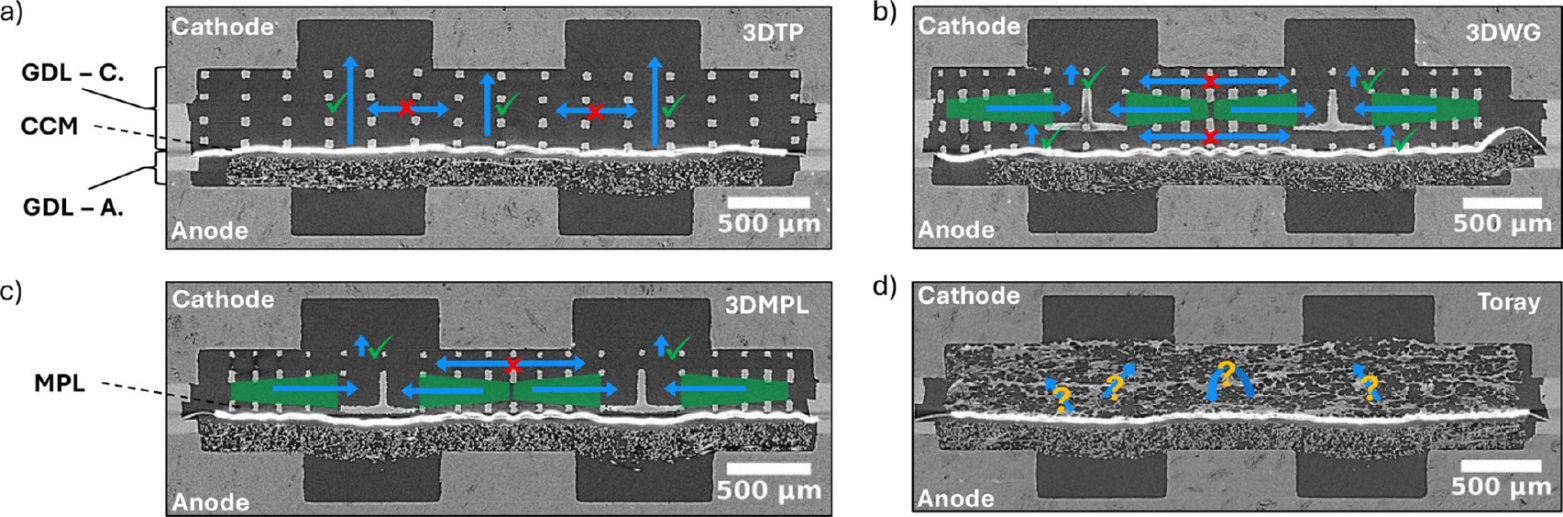
Tomographic slice showing the cross section of a) cell assembly with printed “through-plane” 3DTP GDL at the cathode; b) cell assembly with printed “Water Guide” 3DWG GDL at the cathode; c) cell assembly with printed water guide GDL featuring an MPL 3DMPL at the cathode (later referred to as “Water Guide + MPL”); d) cell assembly with reference Toray GDL at cathode side (later referred to as “Toray”).

Different conceptual designs have been studied in this work, as summarized in Figure 5. The idea of the first structure is to facilitate water percolation only in the direction perpendicular to the CL by designing a 3-layered lattice structure exhibiting through-plane throats with larger constrictions (∼200 μm) than those in the in-plane (∼100 μm) direction,^23^ referred as 3DTP (see Figure 5a). In the second structure, the middle layer is replaced by a guiding layer which features a throat size gradient in the in-plane direction starting with a ∼100 μm throat in the center of the rib increasing toward the channel center to ∼175 μm in 3 steps (∼25 μm each). The objective is to guide the water toward the center of the gas channels no matter at which location it percolates from the first layer, which again only allows through-plane percolation. Once the center of the channel is reached, the water is released through the third layer into the channel with through-plane pores of 200 μm each (see Figure 5b). The in-plane throats of the first and third layers are constant at ∼100 μm. The concept has been already introduced and confirmed by simulation in previous work.^32^ This structure will be referred to as 3DWG. Next, the layer at the GDL-CL interface of the 3DWG is replaced by a free-standing MPL (Figure 5c). MPLs are well-known to improve fuel cell performance by improving water management across various conditions and are therefore indispensable for state-of-the-art PEFC. This design will be referred to as 3DMPL. On the cell assembled with two sandwiched Toray TPH-090 carbon papers highlights the contrast between water percolation pathways of the ordered structures compared to a conventional stochastic GDL material (see Figure 5d).

An overview of the cells tested is given in Table 2. Furthermore, some properties of the commercial GDLs compared to the 3DTP sample as representative for the 3D-printed designs are provided in Table 3. It is found that in terms of permeability, effective diffusivity, as well as porosity, the 3D-printed samples show superior properties compared to the state-of-the-art GDL. However, the electrical conductivity of the carbonized structures is by a factor of five to nine lower than for the Freudenberg or Toray GDLs, respectively.

**Table 2:** Overview of the Nomenclature of the Tested Cells

Cell name	Description
3DTP	3D-printed through-plane
3DWG	3D-printed water guide
3DMPL	3D-printed water guide featuring an MPL
Toray	Reference with two TPH-090 GDLs
Freudenberg	Reference with Freudenberg GDL at anode and cathode

**Table 3:** Characteristic Parameter Comparison of Commercial State of the Art GDLs (TPGH: Toray; FB: Freudenberg) with 3D-Printed Material^a^

Property	TPH-090	FB H2315C2	3DTP
MPL	No	Yes	No
Electr. conductivity (S/m)	15,000^35^	∼8400	∼1600
Permeability (m^–2^)	1.7 × 10^–11^	8.6 × 10^–12^ (GDL)^36^	1.57 × 10^–9^
Effective diffusivity (−)	0.325	0.366 (GDL)^37^	0.81
Porosity (−)	0.7	0.8 (GDL)^38^/0.56 (MPL)^39^	0.88
Thickness (μm)	270	235	500

a The electrical conductivity is in-plane conductivity. Permeability and diffusivities are given for the through-plane direction.

This could be an effect of the lower temperature of the carbonization. Commercial carbon fibers derived from a polyacrylonitrile (PAN) precursor are carbonized at temperatures of 2000–3000 °C, compared to only 1200 °C used in the carbonization of the 3D-printed structures in this study.^2,33^ The electrical conductivity of our structures is already an order of magnitude higher compared to other work^23^ likely due to the higher graphitization temperatures above 1100 °C^34^ rather than the use of a different resin. At a current density of 1 A/cm^2^, the ohmic loss of the 3DTP is ∼3 mV compared to ∼0.3 mV for Freudenberg, which is negligible in comparison to other losses. Furthermore, the thickness of the 3D-printed structures in here is much larger than the conventional GDLs due to printing resolution issues that may be overcome with advancements in 3D printing technology.

### Analysis of Convective GDL In-Flow

**Figure 6:**
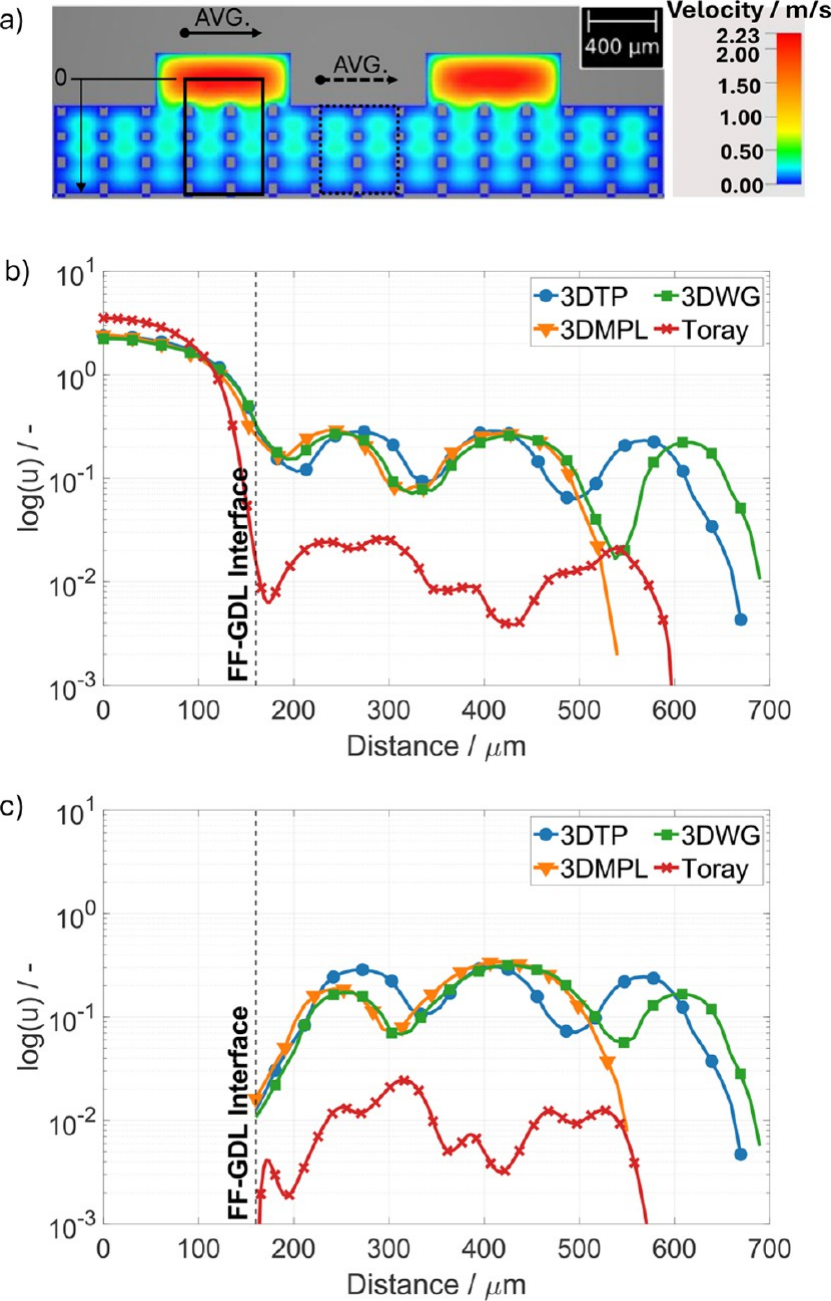
a) Heat map of the estimated velocity distribution (averaged along the channel) exemplarily shown for 3DTP. The boxes qualitatively depict the regions of interest in the channel region (solid) and land region (dashed). b) Through-plane velocity profiles under the channel region. c) Through-plane velocity profiles under the land region. Channel and land regions are highlighted exemplarily by a solid and dashed box in (a), respectively. *X* = 0 in (b) and (c) represents the center of the flow field channel perpendicular to the GDL.

For all the 3D-printed samples, the flow velocity within the GDL is estimated to be at least an order of magnitude higher than in the Toray case, reaching approximately 10% of the peak velocity within the channel even in the lowest layer close to the CL (see Figure 6). Furthermore, no significant difference between the channel and land regions is found (see Figure 6b,c, respectively). The characteristic bumps in Figure 6b,c observed for all 3D-printed structures are related to the horizontal fibers, which basically “break” the flow and therefore exhibit local minima in the flow velocity profile. In contrast, the velocity within the Toray drops by 2 orders of magnitude already close to the GDL-FF interface. It should be noted that the use of the Stokes solver will lead to an overestimation of the flow velocities, especially for the Toray GDL with much smaller pores than the 3D-printed GDLs. The findings suggest that the 3D-printed structures enable enhanced convective reactant transport due to their high porosity and rather large pores and ordered morphology.

### Cell Performance

**Figure 7:**
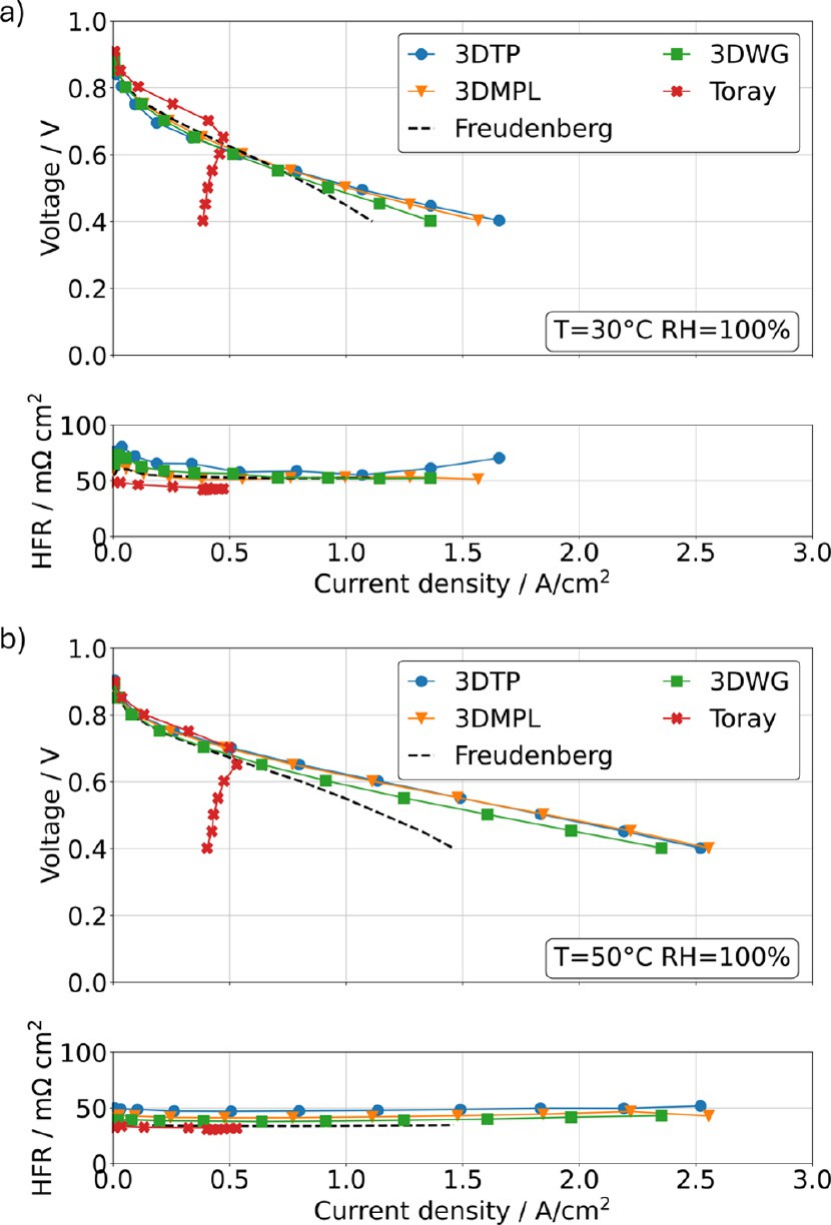
a) Polarization curves and HFR of the tested samples obtained at *T* = 30 °C and RH = 100%. b) Polarization curves and HFR of the tested samples obtained at *T* = 50 °C and RH = 100%.

The polarization curves of all tested cells for performance assessment are shown in Figure 7 for the two temperatures. Note that the active area has been corrected according to the interfaces in touch with both anode and cathode CL, as obtained from the reconstructed tomograms to account for differences in the utilized active area (see Figure S2). The polarization characteristics of the cell with 3D-printed cathode GDL structures with commercial CCMs look much more promising than previously reported results with OCV around 0.6 V^23^ using custom-made CL but thicker membrane. Furthermore, it can be seen that in none of the cells using 3D-printed cathode GDLs the mass transport limitations were reached, which are typically represented by an exponential decay in the high current density region. Instead, they even outperform a commercial Freudenberg carbon fiber GDL with MPL, which performs similar as shown earlier in this cell design.^40^ In contrast, the Toray cell shows limiting current behavior already below 0.5 A/cm^2^. The poor performance of Toray carbon papers at high relative humidities especially at high thicknesses is well-known in literature and therefore expected.^20,41^ The fact that the current density even decreases when going to lower voltages is rationalized by the fact that voltage holds are only 30 s and therefore the polarization curves display a transient behavior. Despite the lower electrical conductivity of the carbonized 3D-printed GDLs, as well as the significantly lower contact area, in particular, between the GDL and flow-field interface, the HFR is in the same order of magnitude for all cells tested. While the differences in electrochemical performance between the 3D-printed GDLs may fall within the error range of repeats, the polarization curve data provide a robust performance assessment of the 3D lattice GDLs. When comparing the 3D-printed samples, the 3DWG falls slightly behind for both tested conditions compared to the 3DTP. In the kinetic region, the Toray paper shows a significantly better performance compared to all other tested materials, especially at lower temperatures. Based on the available measurement data obtained for this study, an explanation for this trend remains ambiguous and beyond the scope of this work. As a side note, the 3DTP cell performed also well in dryer conditions at a temperature of 50 °C and a relative humidity of the supply gases of 60%, though with increased HFR as a result of the drier membrane state (see Figure S3).

### Imaging

X-ray radiography and X-ray tomography have been used to obtain qualitative time-resolved data of the water percolation pathways upon a current jump as well as quantitative 3D steady-state data at the end of the experiment, respectively. The cells’ time-resolved performance during the current jump experiments can be found in Figures S4–S11.

**Figure 8:**
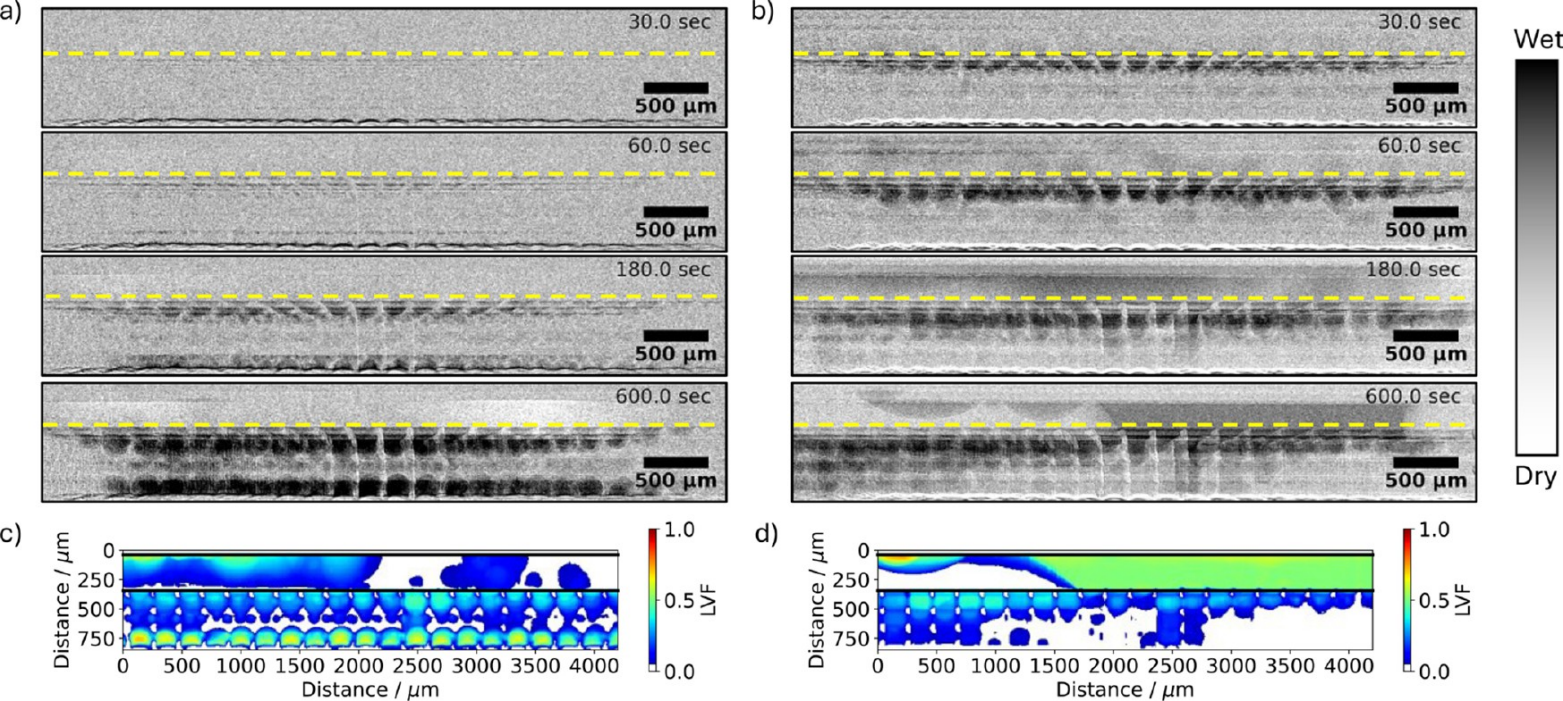
Radiographic difference images (perspective A) of the 3D-printed GDL structure 3DTP 30, 60, 180, and 600 s after the current jump to a) 0.5 A/cm^2^ at *T* = 30 °C and b) 1.5 A/cm^2^ at *T* = 50 °C. The yellow dashed line depicts the GDL-FF interface. Liquid volume fraction obtained from tomographic imaging of the 3D-printed GDL structure 3DTP at the end of the current jump experiment to c) 0.5 A/cm^2^ at *T* = 30 °C and d) 1.5 A/cm^2^ at *T* = 50 °C. The air flow is from right to left in all figures a–d.

The radiography in Figure 8 shows that at low current density and low temperature, the water amount grows almost symmetrically under the land as well as on the CL surface in the 3DTP cathode GDL (see Figure 8a). First liquid water droplets form under the ribs of the flow fields after around 60 s. At 180 s liquid water is found both on the CL as well as under the rib. The bright domain in the channel region after 600 s though suggest that some water slug that has been already there at the start of the experiment has been removed, which can be either due to insufficient drying of the gas channel or water condensation being transported with the air flow entering the channel just at the beginning of the experiment. In contrast, at the high current density and high temperature conditions, water seems to be transported exclusively in vapor from the CL surface and eventually condensates under the ribs (see time series in Figure 8b). The very same trend between the two tested conditions is observed to be similar for all tested 3D-printed structures. Only the Toray reference cell shows liquid water evolution on the GDL-CL interface only, in both conditions. Animated videos of the difference image of all tested structures, conditions, and perspectives can be found in Videos S1–S16. The animated radiography data of the high current density condition further reveal several events in which large amounts of water are drained from the GDL when liquid water is pushed through the channel (see Video S3 at seconds: 162, 192, 412, and 515). Further examples can be found for all of the tested 3D-printed structures across all conditions. This suggests a strong advective water transport likely due to the ordered structure and associated high connectivity of the water within the GDL. Furthermore, the LVF of the low and high temperature cases obtained from tomographic imaging after each experiment are shown for the 3DTP in Figure 8c,d, respectively. The LVF confirms the observations obtained from the radiography. In the case of the low temperature and current density, more water is present at the CCM surface as compared to the ribs (see Figure 8c). Note that the LVF should not be mistaken for the saturation as it does not consider the solid material fraction.

Other than previous findings it seems here we are encountering a mixed transport scenario of liquid and vapor phase transport at 30 °C and only vapor transport at 50 °C with the relative humidity being 100% in both cases.^10^ It needs to be considered that the waste heat produced in the electrochemical reaction leads to a temperature gradient across the GDL with a higher temperature close to the CL. This effect is stronger in the high current density case due to the higher amount of heat produced. These observations together with the flow distribution obtained from the simulation suggest that at 50 °C operation the water is predominantly produced in vapor form and transported by either convection or diffusion toward the colder spots under the ribs, where it partially condensates. Mularczyk et al. investigated on the evaporation of water in GDL and found that with increasing relative humidity of the gases the evaporation limit shifts from diffusion limited to convection limited.^42^ Considering the high relative humidity conditions explored in this study, it is expected that convection determines the evaporation limit introduced by the rather high gas velocities close to the CL as shown in Figure 6.

**Figure 9:**
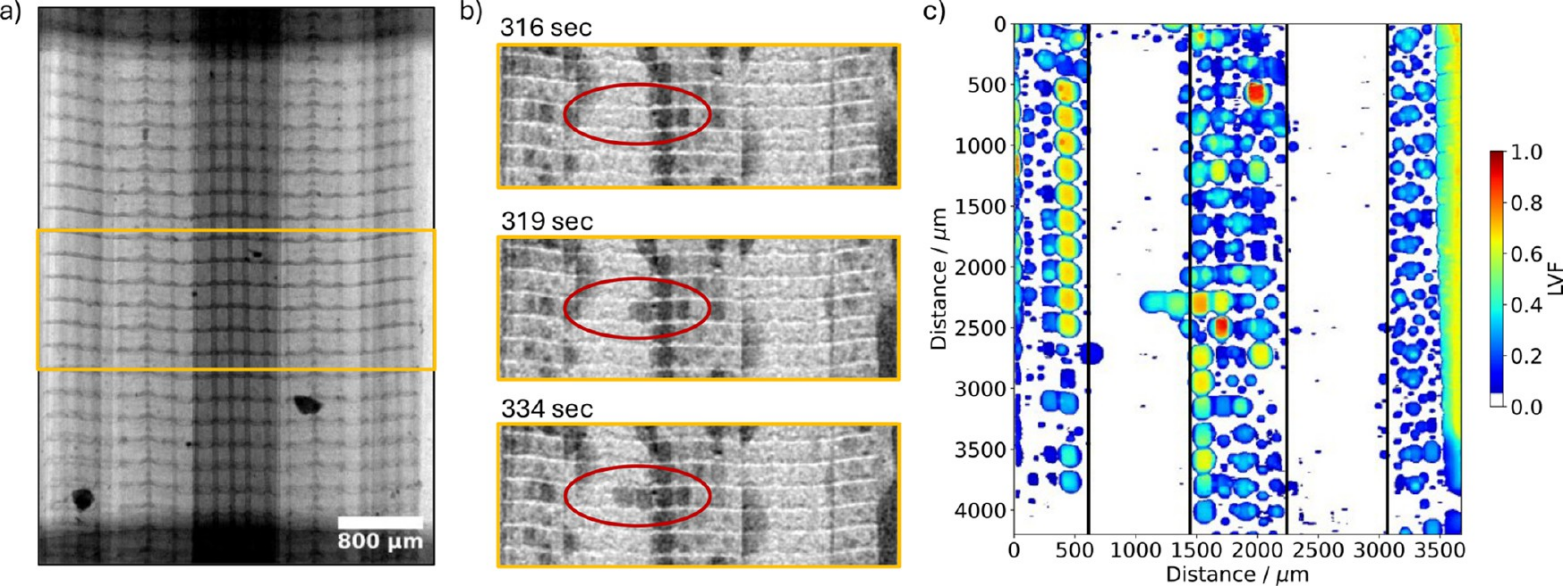
a) Radiographic projection (perspective B) of the dry state of 3DWG. b) Three timesteps showing the difference image of the region depicted in (a) for the current jump to 1.5 A/cm^2^ at *T* = 50 °C, scale bar same as in (a). c) In-plane view of the liquid volume fraction of the cell showing the in-plane cross-section after the current jump for perspective A.

Next the 3DWG is investigated with respect to the water transport in the channel-rib region to confirm that the introduced guiding layer functions as intended (Figure 9). The raw image of the dry cell before the current jump is shown in Figure 9a. The difference images in Figure 9b for time steps 316, 319, and 334 s after the current jump for the region depicted in the orange box in Figure 9a shows a capillary finger growing perpendicular to the rib. Further proof can be found in the corresponding videos (Videos S6, S8, and S10). The initial objective of guiding the water toward the center of the channel followed by breakthrough into the channel lead to another effect: when liquid water is flushing through the gas channels it seems to drag water from under the rib as the snap off location is only under the center of the rib, instead of randomly and eventually near the gas channel in the case of conventional GDLs. Similar to the observations of the radiography, the tomographic image exhibits a capillary finger trapped in the pores under the left channel located at *y* = 2300 μm (see Figure 9c). Furthermore, the LVF reveals a very small amount of water, especially in the channel region where almost no water is found. Note that the LVF for the in-plane view only shows water within the GDL and channels have not been included.

**Figure 10:**
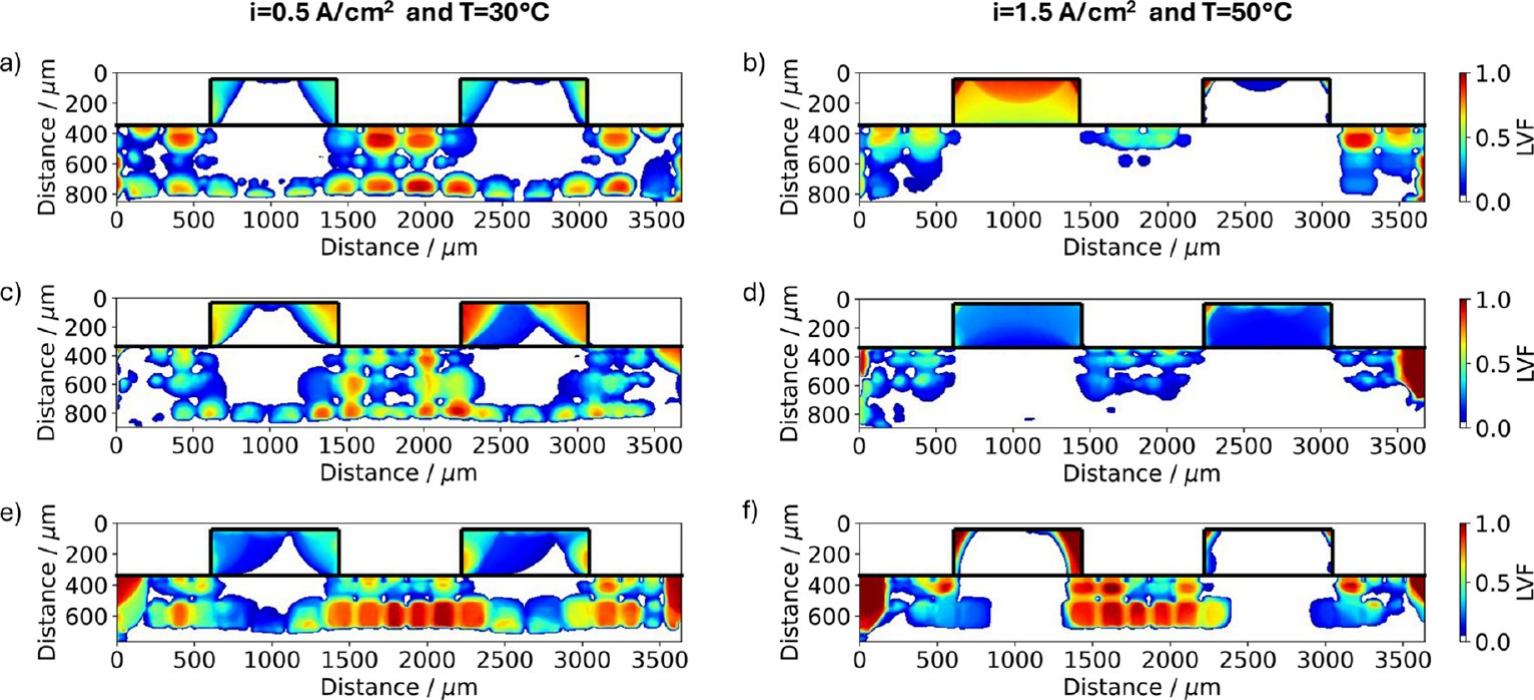
Liquid volume fraction obtained from tomographic imaging at the end of the current jump experiment showing the cross-sectional view of the GDL at the different conditions as labeled on top of the figure for (a, b) 3DTP, (c, d) 3DWG, and (e, f) 3DMPL.

**Figure 11:**
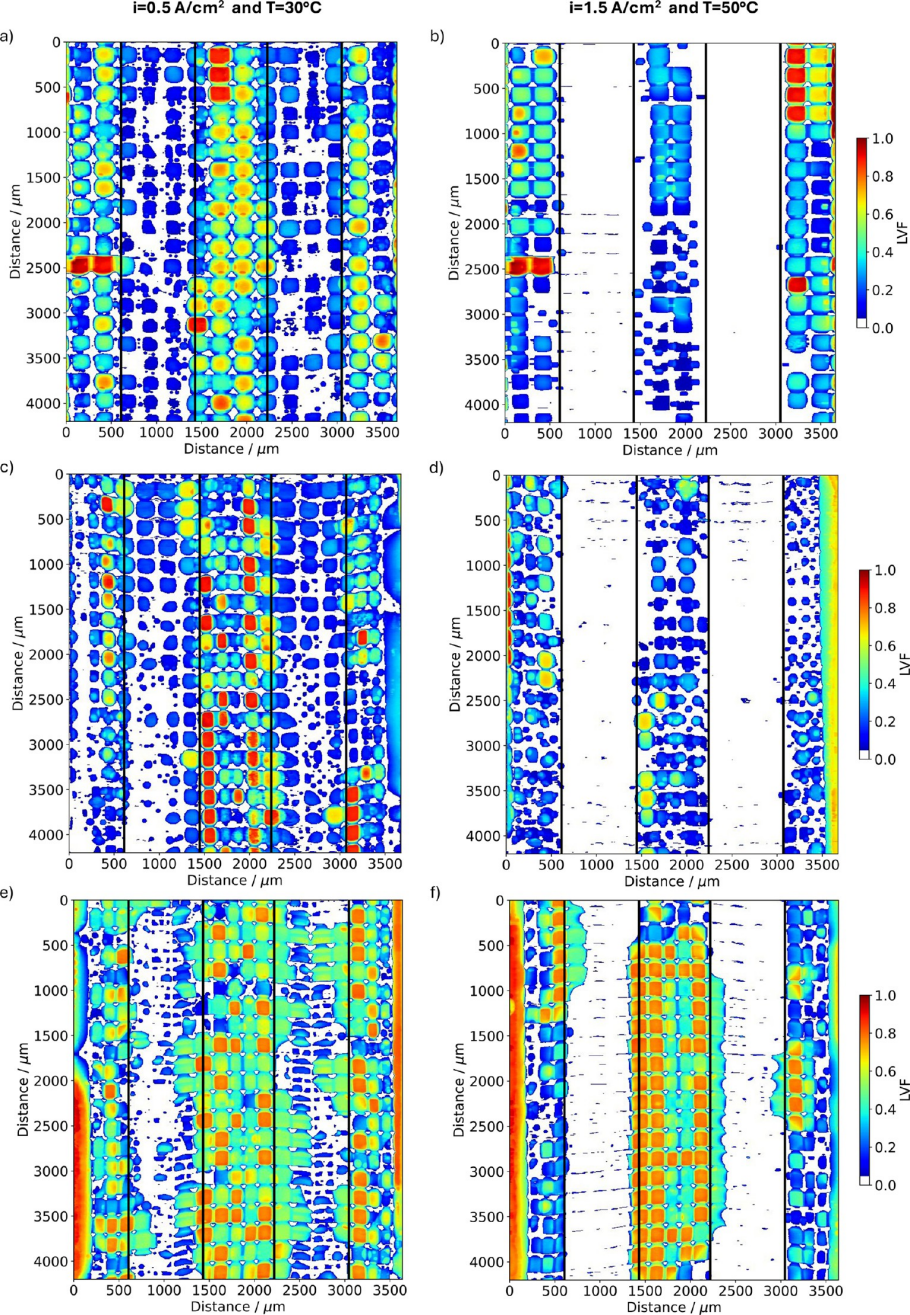
Liquid volume fraction obtained from tomographic imaging at the end of the current jump experiment showing the in-plane view of the GDL at the different conditions as labeled on top of the figure for (a, b) 3DTP, (c, d) 3DWG, and (e, f) 3DMPL.

To get a more comprehensive understanding of the differences between the investigated structures and conditions, the LVF for the cross-sectional view parallel to the gas flow is shown in Figure 10 for the low and high current density conditions. Furthermore, the corresponding LVFs in the in-plane view are shown in Figure 11 for both conditions. Here, the distinct three-layer design enables direct interpretation of the number of layers filled based on LVF values (red = three layers, orange = two layers, blue = one layer). It should be mentioned that for the 3DMPL, the first layer corresponds to the MPL, which remains unresolved in the imaging due to the much smaller pores of the MPL.

When looking into Figure 10, it can be observed that the LVF in the channel region is much lower than in the land region for all 3D-printed GDLs. It looks like there is an “extension” of the channel reaching into the GDL which is a consequence of the convective flow within the structure. It is further evident that both samples with the guiding layer, 3DWG and 3DMPL, exhibit a lower LVF close under the rib compared to the 3DTP (see low LVFs at [*x* = 1600–1900 μm | *y* = 500–700 μm] in Figure 10c and [*x* = 1800–2000 μm | *y* = 350–500 μm] in Figure 10e). This reduction can be attributed to the effective function of the guiding layer. In the case of the 3DMPL, the LVF appears much larger compared to the other cells. However, this structure is significantly thinner than the 3DTP and 3DWG due to the missing first layer and therefore has a lower capacity to store liquid water which effectively increases the LVF. At low current density (*T* = 30 °C), the 3DTP exhibits only four fully saturated spots under the central rib (*x* = 1450–2200 μm), while the 3DWG and 3DMPL samples have 22 and 30 entirely filled sites, respectively (see red zones in Figure 11a,c and orange zones in Figure 11e, respectively). The 3DMPL sample shows a more heterogeneous water distribution and in general a higher amount of liquid water in the channel region, whereas for the 3DTP and 3DWG less water is observed and rather homogeneously distributed next to the CL (Figures 10a,c,e and 11a,c,e).

When further looking into the high current and temperature condition, one can observe a clear shift of the LVF from the CL toward the land region. This is particularly prominent for the 3DTP as well as the 3DWG GDLs where almost no water is found close to the CL (see Figure 10b,d as well as Figure 11b,d). For the 3DMPL, this trend is less pronounced, and only the region under the channel is found to be dry, while in the land region a lot of water is present. Similar to the low current density case, a region of reduced LVF can be identified in the very center under the rib (see [*x* = 1700–2000 μm/*y* = 350–700 μm] in Figure 10f). This, along with the water located in the rib-neighboring pores in the second layer (at [*x* = 600–800 μm, *x* = 2200–2400 μm and *x* = 2900–3100 μm | *y* = 500–700 μm]) can once again be ascribed to the impact of the GDL design on forced water percolation. The only fully flooded region observed in the 3DTP sample at [*x* = 0–400 μm | *y* = 2500 μm] in Figure 11a,b corresponds to a crack in the GDL (Figure S12). The 3DWG sample shows more water near the inlet (*y* = 2000–4200 μm), distributed across the full rib width (*x* = 1450–2200 μm), but a lower LVF near the outlet (*y* = 0–2000 μm; see Figure 11d). In the corresponding radiography (Video S8), it can be observed that after 220 s there is always at least one of the channels blocked by a water slug which leads to an increased convective GDL in-flow. It can be also seen that at the moment where the water enters the channel the liquid water under rib decreases and remains low compared to the situation before 220 s throughout the entire experiment. Compared to the 3DWG, the 3DMPL holds significantly more water under the rib, with some even reaching into domains under the channel (*x* = 1400–1450 and 2200–2250 μm in Figure 11f, see also Figure 10f). Unlike the 3DMPL, the channel regions in 3DTP and 3DWG remain almost entirely dry under these conditions. It has been reported earlier that the thermal conductivity of MPLs is rather low compared to the substrate.^43^ It is hypothesized that the MPL and thinner GDL lead to a significantly lower temperature on the MPL-GDL interface compared to the CL-GDL interface for the 3DTP and 3DWG which results in a more saturated environment, and therefore, water vapor is more likely to condensate. Additionally, the 3DMPL displays a defined domain of lower LVF in the rib center in Figure 11f at *y* = 1700–2000 μm, consistent with observations from the 2D channel-rib distributions shown in Figure 10f. The effect of a blocked channel is also observed for the repeat of the current jump to 1.5 A/cm^2^ at *T* = 50 °C for 3DMPL. In the corresponding radiography (Video S11) one of the channels is entirely flooded at the start of the experiment until 138 s and then again from 400 s until the end of the experiment, as also captured in the tomographic image set (see LVF in Figure S13a). In this case, the LVF under the center rib is shown in Figure S13a,b is significantly lower as compared to the “twin” that was discussed before in Figures 10f and 11f, respectively. The reduced LVF again suggests a significantly increased convective gas flow within the GDL due to the blocked channel. For completeness, the LVF from the side view are shown in Figure S14.

The LVFs for the Toray cell can be found in Figures S15 and S16. Contrary to all of the 3D-printed samples, more water is observed in the channel region with an LVF around 0.5. Previous studies have shown that in very wet conditions and high current densities the current distribution at the CL shifts toward regions under the channel.^44^ This explains the bad performance of the Toray material as the liquid water close to the CL blocks the oxygen pathways toward the active sites. In fact, it is well-known that the porosity of carbon papers with a hydrophobic treatment is particularly low at the surfaces (∼60%) as PTFE tends to accumulate.^15,45^

### Summary and Conclusion

In this study, GDL structures with a deterministic pore space based on cubic lattices have been successfully printed, carbonized, and tested in a fuel cell setup designed for X-ray imaging. An alternative hydrophobic treatment has been used to achieve homogeneous wetting properties compared to conventional PTFE coatings, which often result in uneven wettability patterns. Three different 3D-printed GDL designs with a deterministic pore space, namely the 3DTP structure for through-plane transport, the 3DWG design, intended to guide water toward the center of the flow field channel and the 3DMPL combining the 3DWG with a free-standing MPL, have been tested.

Compared to commercial GDLs, the 3D-printed designs exhibit higher porosity as well as permeability and diffusivity, as shown by numerical simulation. However, the electrical conductivity is lower, which is expected to be a result of the lower temperature during the carbonization process. Flow simulations further revealed a significantly increased convective flow within the 3D-printed structures mounted in a cell, an order of magnitude larger than that in a state-of-the-art carbon paper. The flow distribution in the GDL is predicted to be homogeneous without showing significant differences between regions under the channel and rib.

The performance of the GDLs was assessed under different conditions. The results showed that the printed GDLs, unlike the reference Toray paper (no MPL) and Freudenberg GDL (with MPL), displayed no significant mass transfer limitations using fully humidified feed gases at 30 and 50 °C, suggesting enhanced water as well as reactant transport properties of the 3D-printed structures. The poor performance of Toray paper without MPL under high humidity conditions is well-documented and is attributed to a liquid layer forming on the CL surface, hindering the oxygen transport in regions under the channel, as confirmed by this work.

The water transport was investigated by operando X-ray radiography as well as X-ray tomography. It was shown for the 3D-printed GDLs, that at low current densities (<1 A/cm^2^), water grows rather symmetrically below the land and on the CL surface, whereas at higher current densities (>1 A/cm^2^) and temperatures (50 °C), water is forming preferentially by condensation under the ribs. This indicates that the heat generation due to the reaction results in a temperature gradient within the GDL, promoting vapor phase water transport away from the CL. The GDL inflow leads to a lower amount of liquid water close to the CL and an improved convective reactant supply, which explains the mass transport limitation-free performance. Furthermore, traces of the desired water percolation pathways have been observed in the guiding layer of the 3DWG and 3DMPL which proves the conceptual idea of guiding water through the structure by adjusting the throat sizes.

**Figure 12:**
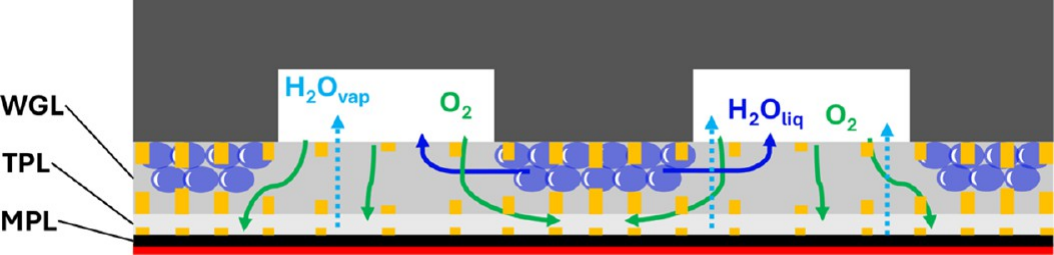
Design proposal for advanced GDL featuring an MPL, a through-plane guiding layer (TPL), and a water guiding layer (WGL). The throat structure and CL are colored orange and red, respectively.

Finally, we propose an improved water guide design, as shown in Figure 12 that considers the mixed vapor and liquid water transport at operating temperatures clearly above room temperature. The objective is to force in-plane percolation of the condensed water under the ribs toward the channels instead of through-plane percolation toward the CL and thereby maintaining intermediate dry layer(s) free of liquid water for highly efficient convective reactant supply in combination with an MPL.

The findings of this study demonstrate the potential of deterministically structured GDLs with designed percolation pathways to enhance fuel cell performance, primarily through improved convective flow within the GDL, which facilitates both efficient water management and reactant supply under high relative humidities of the supply gases. While the ideal design of the pore space can still be optimized, the results indicate the need for incorporating an MPL to mitigate membrane dryout and associated high ohmic resistance under low humidity conditions. Moreover, the findings also suggest that these highly porous structures eliminate the need for channels in general and offer the potential to also serve as porous flow fields. However, realizing the potential of these optimized structures on a larger scale depends on the advancement of cost-effective high-resolution printing technologies capable of printing such structures at a larger scale. Future work should focus on comprehensive performance assessments and larger, elongated active areas, particularly under drier operating conditions, to fully explore the practical feasibility and advantages of the deterministic GDL with high porosities.

## Supporting Information Available

Video S1: Animated radiography time series of 3DTP, perspective A, *T* = 30 °C (MP4)
Video S2: Animated radiography time series of 3DTP, perspective B, *T* = 30 °C (MP4)
Video S3: Animated radiography time series of 3DTP, perspective A, *T* = 50 °C (MP4)
Video S4: Animated radiography time series of 3DTP, perspective B, *T* = 50 °C (MP4)
Video S5: Animated radiography time series of 3DWG, perspective A, *T* = 30 °C (MP4)
Video S6: Animated radiography time series of 3DWG, perspective B, *T* = 30 °C (MP4)
Video S7: Animated radiography time series of 3DWG, perspective A, *T* = 50 °C (MP4)
Video S8: Animated radiography time series of 3DWG, perspective B, *T* = 50 °C (MP4)
Video S9: Animated radiography time series of 3DMPL, perspective A, *T* = 30 °C (MP4)
Video S10: Animated radiography time series of 3DMPL, perspective B, *T* = 30 °C (MP4)
Video S11: Animated radiography time series of 3DMPL, perspective A, *T* = 50 °C (MP4)
Video S12: Animated radiography time series of 3DMPL, perspective B, *T* = 50 °C (MP4)
Video S13: Animated radiography time series of Toray, perspective A, *T* = 30 °C (MP4)
Video S14: Animated radiography time series of Toray, perspective B, *T* = 30 °C (MP4)
Video S15: Animated radiography time series of Toray, perspective A, *T* = 50 °C (MP4)
Video S16: Animated radiography time series of Toray, perspective B, *T* = 50 °C (MP4)
Resin composition, additional cross-sectional SEM image, correction of the cells active area, electrochemical data during current jump experiments, cracks in GDL observed by X-ray tomography, additional LVF maps (PDF)
