# Supplementary material for: Guided Water Percolation in 3D-Printed Gas Diffusion Layers for Polymer Electrolyte Fuel Cells
Source: ACS Appl Mater Interfaces. 2025 Apr 10;17(16):23959–71. doi: 10.1021/acsami.5c00770 (PMC12022989; doi:10.1021/acsami.5c00770)
Supplement: Supplementary file 17 — am5c00770_si_017.pdf [file am5c00770_si_017.pdf]

Supporting information
Guided Water Percolation in 3D-Printed Gas Diffusion Layers for Polymer Electrolyte Fuel Cells

Tim Dörenkamp[1], Ambra Zaccarelli[2], Felix N. Büchi[1], Thomas J. Schmidt[1,3] and Jens Eller[1,]*

[1]PSI Center for Energy and Environmental Sciences, CH-5232 Villigen PSI, Switzerland
[2]Department of Materials, ETH Zürich, CH-8093 Zürich, Switzerland
[3]Institute of Molecular Physical Science, ETH Zürich, CH-8093 Zürich, Switzerland

* Corresponding author: jens.eller@psi.ch

Resin composition

Table S1. Formula of the Phrozen Aqua Grey 8K Resin obtained from the data sheet.

**Components**	**CAS number**	**Weight**
Dipropylene Glycol Diacrylate	Proprietary	Proprietary
Epoxy acrylate	Proprietary	Proprietary
Acryloyl morpholine	Proprietary	Proprietary
Tris(2-Hydroxy Ethyl) Isocyanurate Triacrylate	Proprietary	Proprietary
Urethane acrylate	Proprietary	Proprietary
Silicon dioxide	Proprietary	Proprietary
Diphenyl(2,4,6-trimethyl benzoyl) phosphine oxide	75980-60-8	1-10 %
Titanium dioxide	13463-67-7	<1 %
Carbon black	1333-86-4	<1 %

Cross-sectional SEM of the carbonized structure

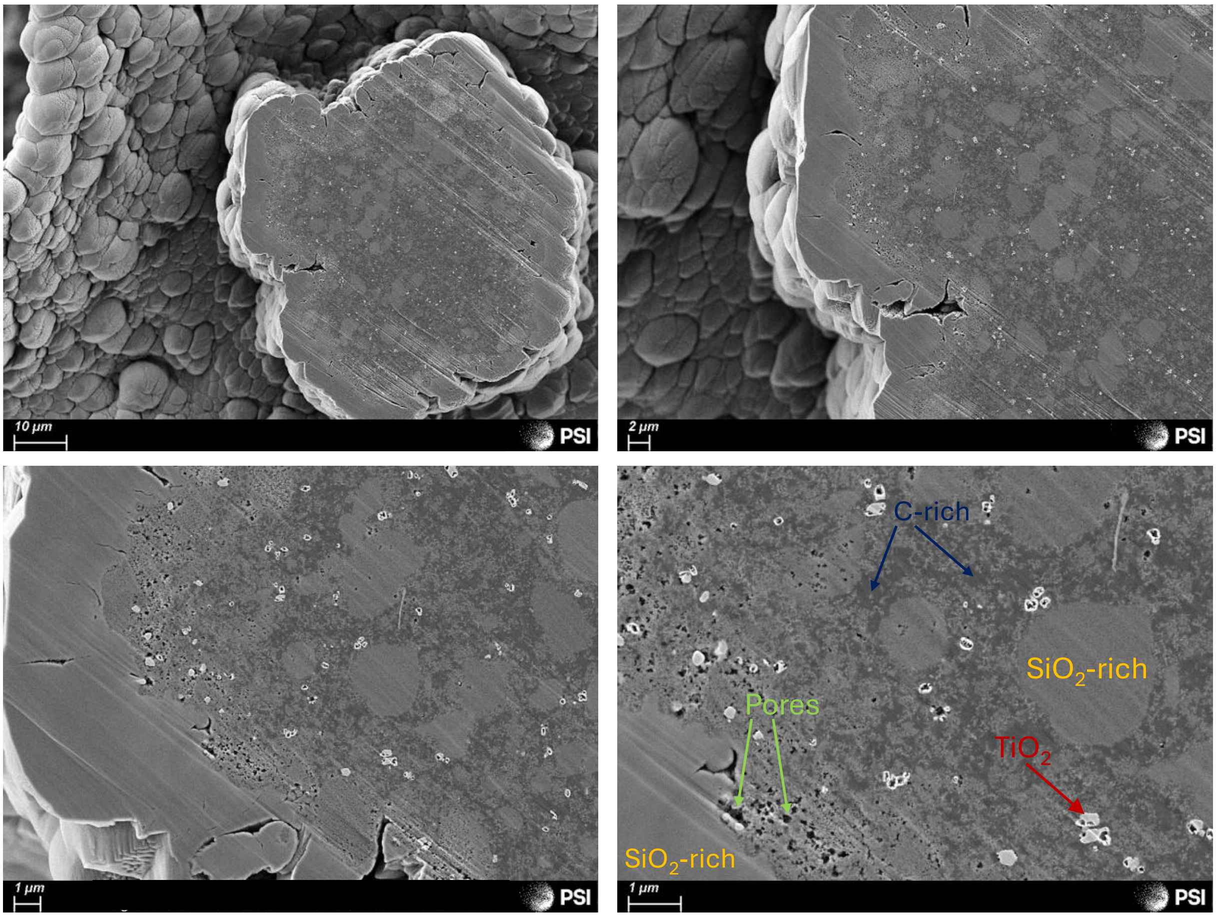

Figure S1. Cross-sectional SEM images of the carbonized structure at varying resolutions. In the bottom right image the composition of the different phases, as determined by EDS measurements, is indicated.

Correction of the active domain

The active area is the part of the CCM which has contact to both anode and cathode GDL. Therefore, the corrected active area can be assumed to be the theoretical active area (0.16 cm^2) times a correction factor obtained from tomographic imaging as shown in Figure S2a:

$$\text{Corr. active area} = \frac{L_{\text{active}}}{L_{\text{total}}} \cdot 0.16\text{cm}^2 \quad \text{S1}$$

The results are listed in Figure S2b.

a)

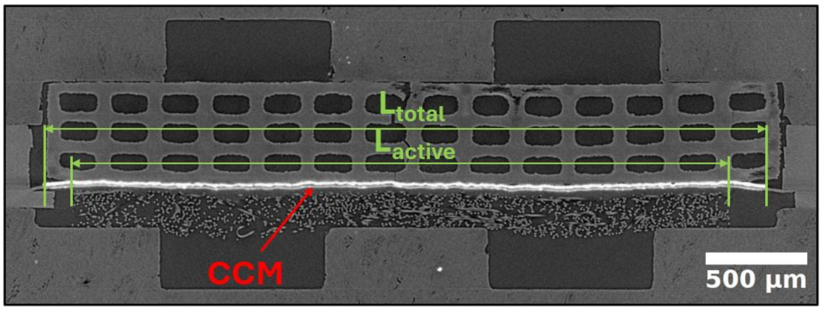

b)

Cell name	Corr. active area / cm^2
3DTP	0.149
3DWG	0.144
3DMPL	0.156
Toray	0.16
Freuden-berg	0.156

Figure S2. a) Cross-section of the 3DTP cell exemplarily depicting the considered active width (Lactive) compared to the total width (Ltotal) of the active area. b) Corrected active areas for the tested cells.

Electrochemical data during current jump Experiments

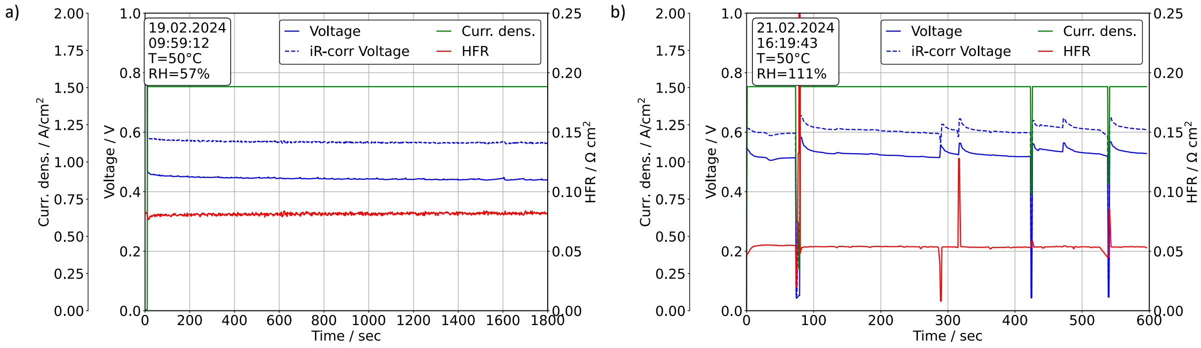

Figure S3. Performance data of the current jump at T=50 °C and i = 1.5 A/cm2 of the 3DTP for a) RHset=60 % and b) RHset=110 %.

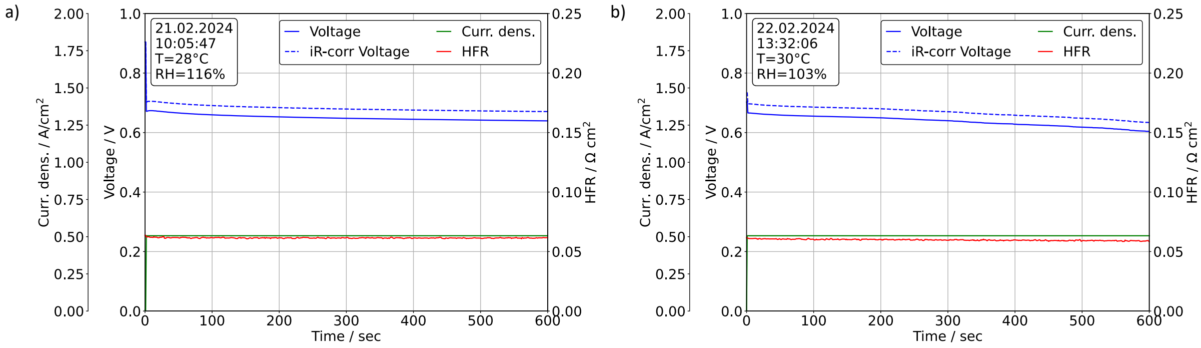

Figure S4. Performance data of the current jump at T=30 °C and i = 0.5 A/cm2 of the 3DTP for a) perspective A and b) perspective B.

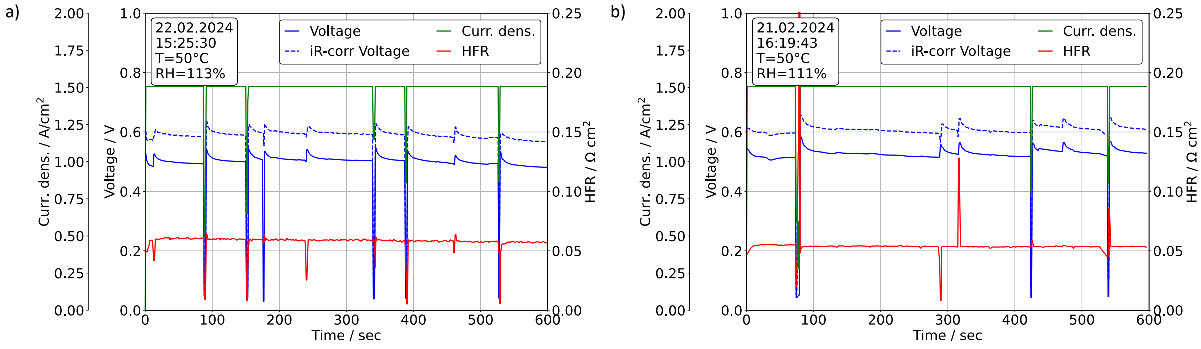

Figure S5. Performance data of the current jump at T=50 °C and i = 1.5 A/cm2 of the 3DTP for a) perspective A and b) perspective B.

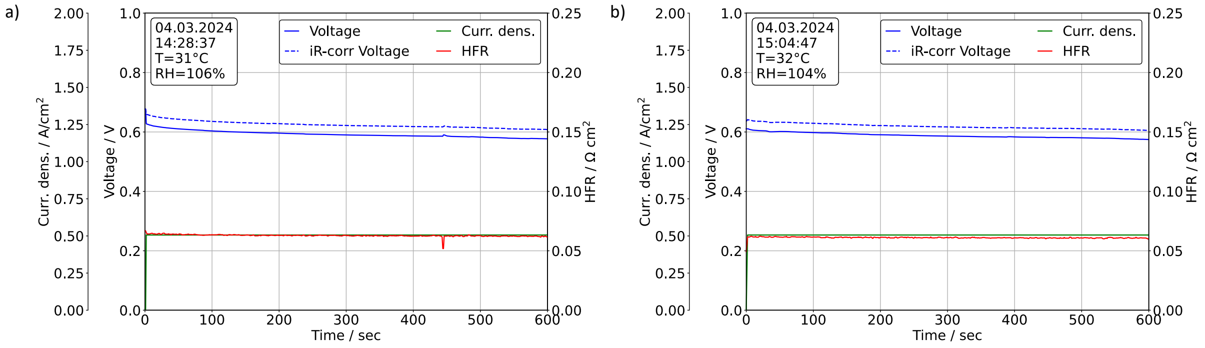

Figure S6. Performance data of the current jump at T=30 °C and i = 0.5 A/cm2 of the 3DWG for a) perspective A and b) perspective B.

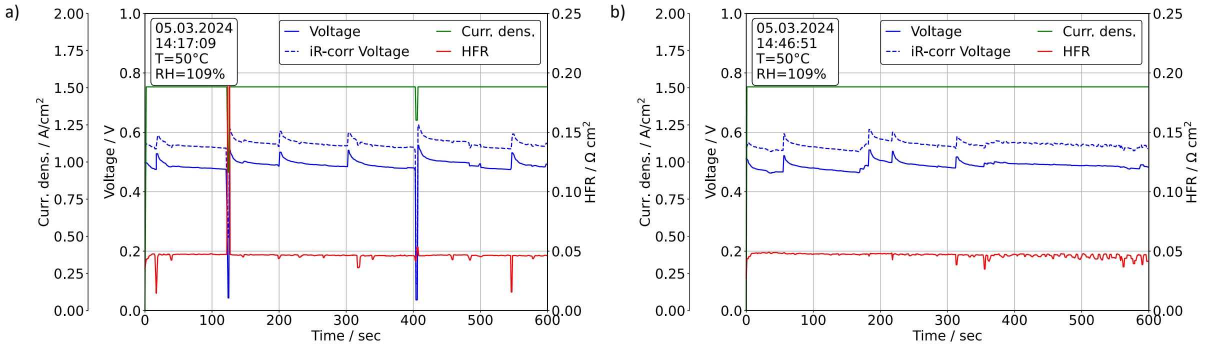

Figure S7. Performance data of the current jump at T=50 °C and i = 1.5 A/cm2 of the 3DWG for a) perspective A and b) perspective B.

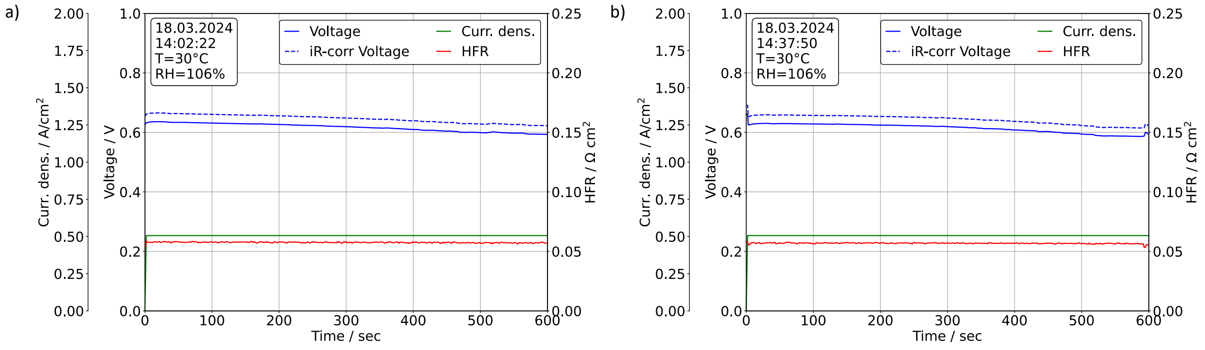

Figure S8. Performance data of the current jump at T=30 °C and i = 0.5 A/cm2 of the 3DMPL for a) perspective A and b) perspective B.

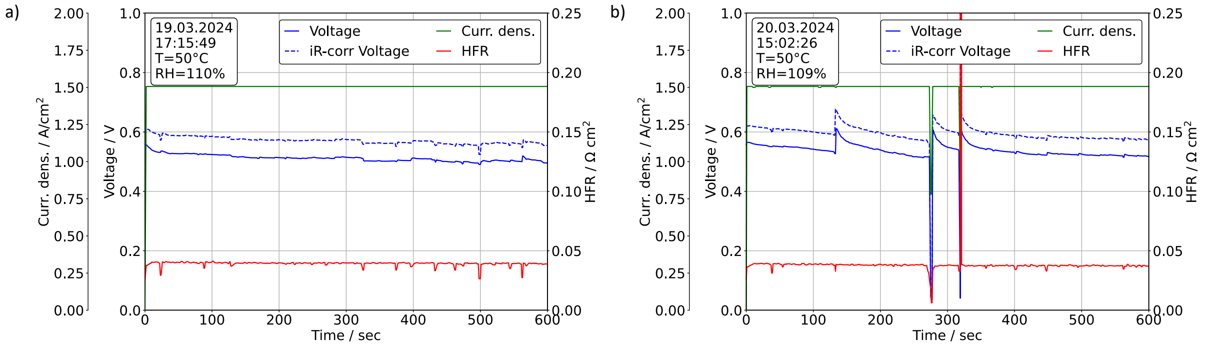

Figure S9. Performance data of the current jump at T=50 °C and i = 1.5 A/cm2 of the 3DMPL for a) perspective A and b) perspective B.

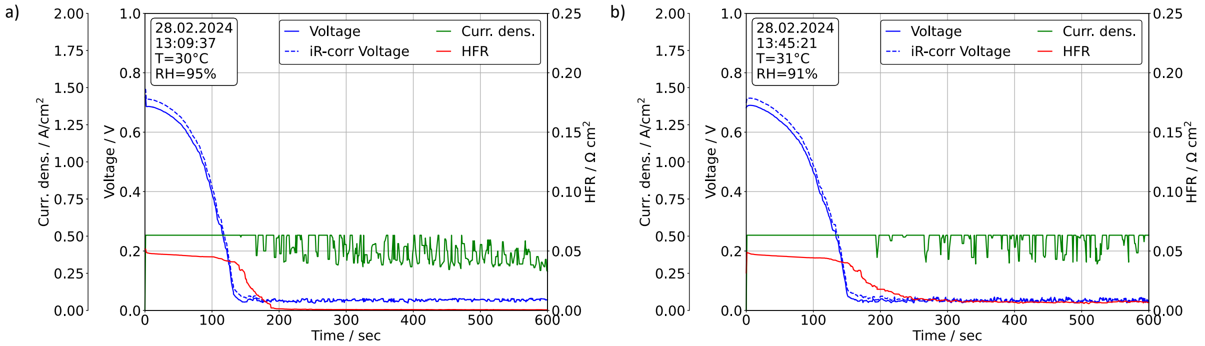

Figure S10. Performance data of the current jump at T=30 °C and i = 0.5 A/cm2 of the Toray for a) perspective A and b) perspective B.

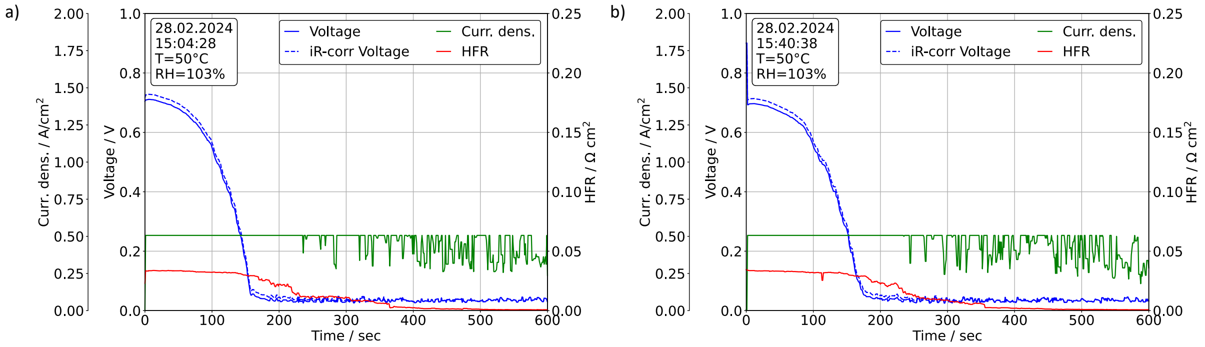

Figure S11. Performance data of the current jump at T=50 °C and i = 1.5 A/cm2 of the Toray for a) perspective A and b) perspective B.

Crack in 3DTP GDL

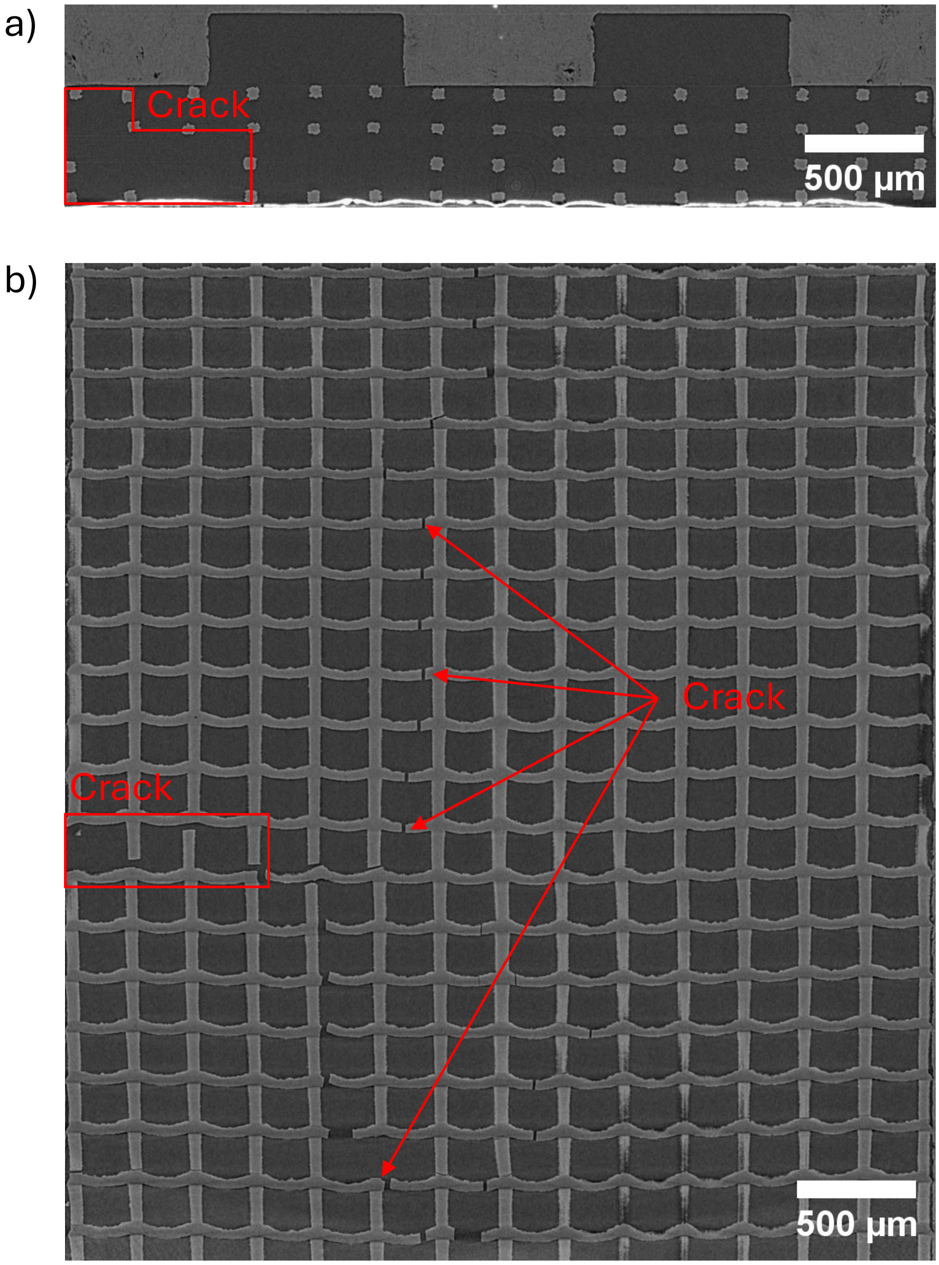

Figure S12. a) Cross sectional view showing cracked GDL of the 3DTP b) In-plane view showing the same crack.

Additional LVF maps

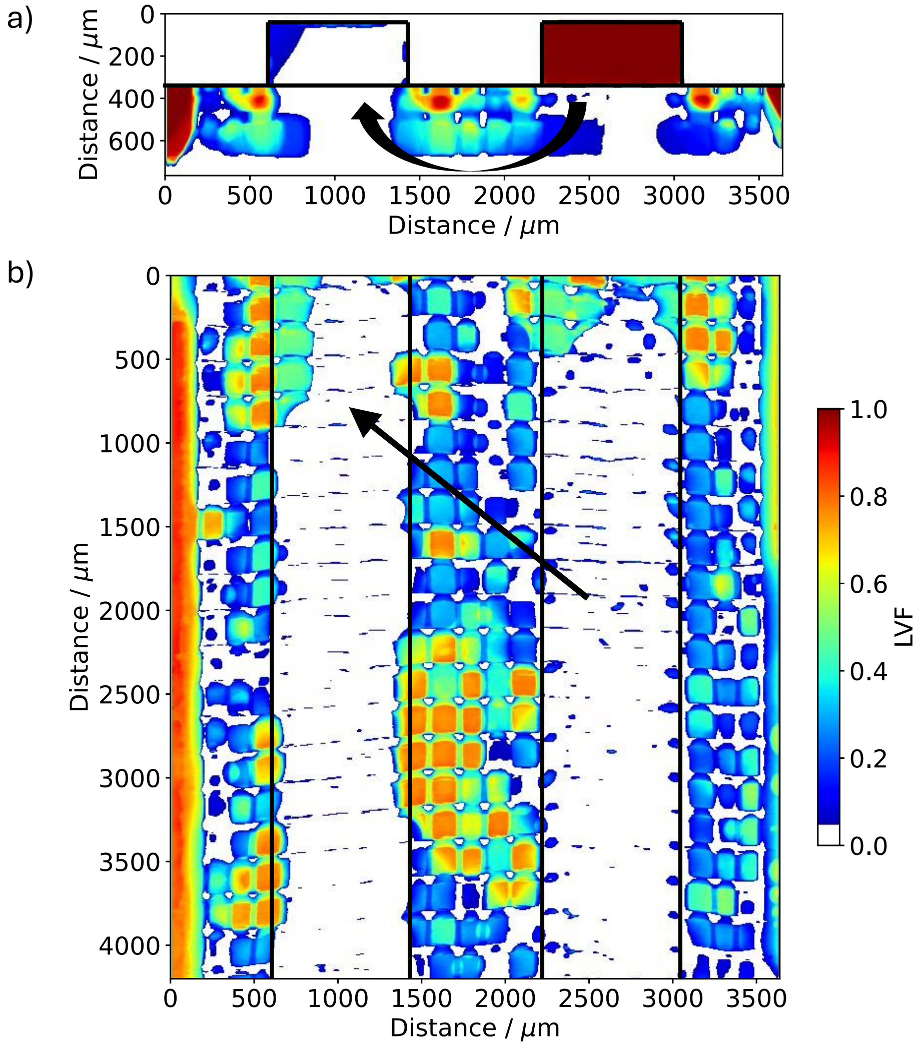

Figure S13. Liquid volume fraction obtained from tomographic imaging at the end of the current jump experiment to 1.5 A/cm2 at T=50 °C for the 3DMPL showing a) the cross-sectional view of the GDL and channels and b) the in-plane view of the GDL. Black arrows depict the suspected direction of crossflow.

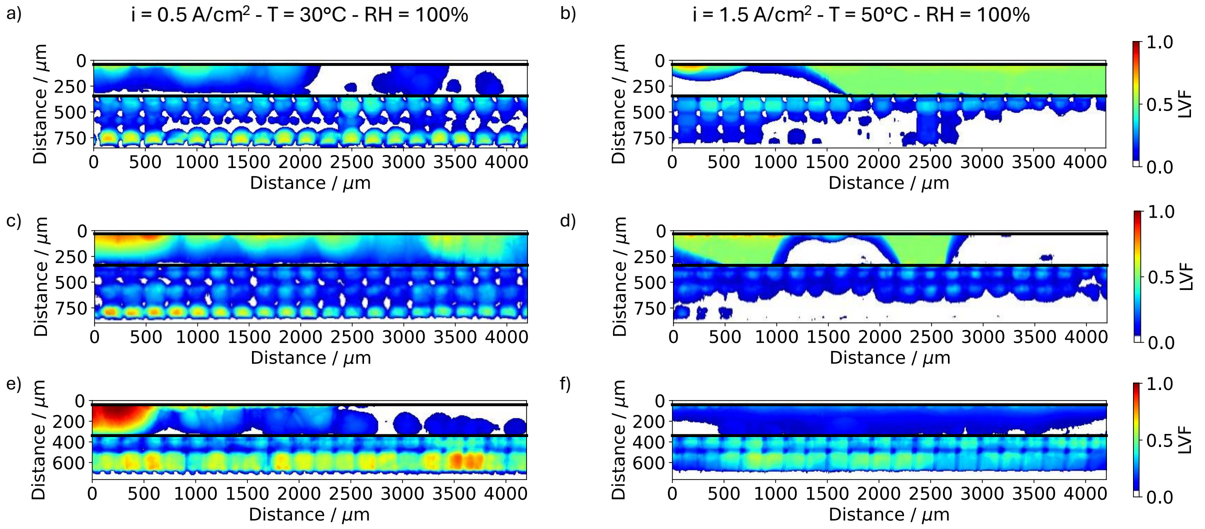

Figure S14. Liquid volume fraction obtained from tomographic imaging in the side view for the 3DTP (a,b), 3DWG (c,d) and 3DMPL (e,f). Left column represents low current density and temperature condition, right column high current density and temperature.

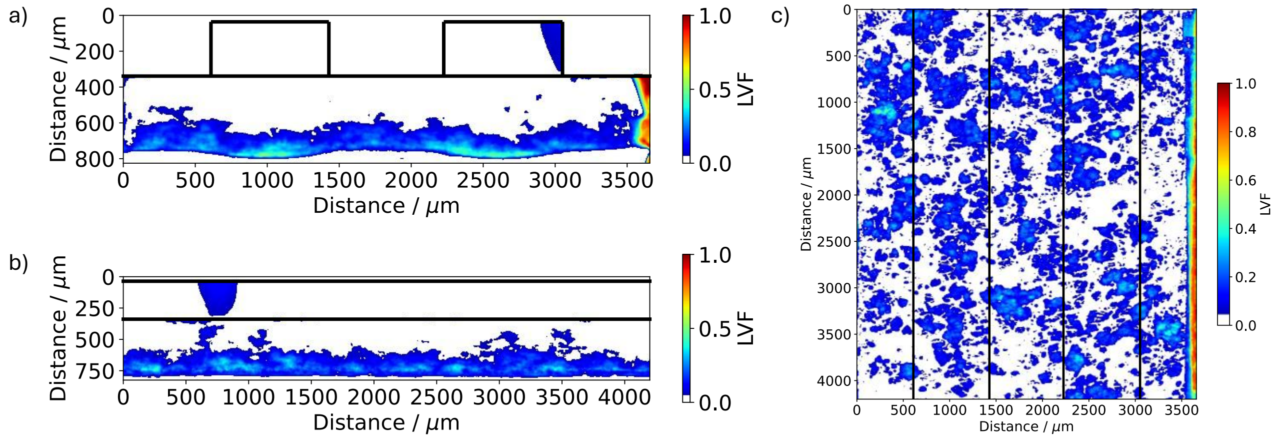

Figure S15. Liquid volume fraction obtained from tomographic imaging at the end of the current jump experiment to 0.5 A/cm2 at T=30 °C for the Toray cell: a) cross section b) side view c) in-plane view

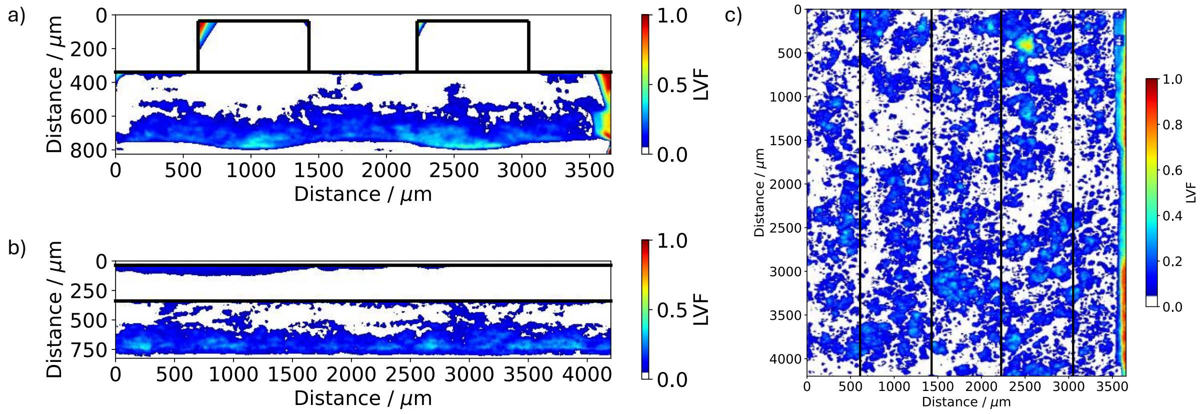

Figure S16. Liquid volume fraction obtained from tomographic imaging at the end of the current jump experiment to 1.5 A/cm2 at T=50 °C for the Toray cell: a) cross section b) side view c) in-plane view
